# Pezizales in Israel: Molecular Phylogenetic and δ^13^Cδ^15^N Stable Isotope Data Reveal New Records and Potential Discrepancies in Their Trophic Ecology

**DOI:** 10.3390/jof11060414

**Published:** 2025-05-27

**Authors:** Segula Masaphy, Gregory Bonito, Ezra Orlofsky, Judson Van Wyk, Benjamin Lemmond, Rosanne Healy, Matthew E. Smith, Yaniv Segal, Limor Zabari

**Affiliations:** 1 Department of Food Sciences, Faculty of Sciences and Technology, Tel Hai Academic College, Kiryat Shmona 12210, Israel; 2 Applied Mycology and Microbiology, Migal, Tarshish 1, Kiryat Shmona 11016, Israel; ezorlo@gmail.com (E.O.); limor@migal.org.il (L.Z.); 3 Department of Plant, Soil and Microbial Sciences, Michigan State University, East Lansing, MI 48824, USA; bonito@msu.edu (G.B.); vanwykju@msu.edu (J.V.W.); 4 Department of Plant Pathology, University of Florida, Gainesville, FL 32611, USA; benlemmond@gmail.com (B.L.); rosanne.healy@gmail.com (R.H.); trufflesmith@ufl.edu (M.E.S.); 5 Independent Researcher, Abirim 13806, Israel; waysoffungi@gmail.com

**Keywords:** ectomycorrhizal, isotopic signature, Pezizales, trophic state, saprotroph, wild mushroom

## Abstract

The order Pezizales (Ascomycota) consists of cup and truffle fungi growing in a wide range of habitats and geographical zones worldwide, exhibiting diverse nutritional behaviors. In Israel, morphological studies have designated most Pezizales as saprotrophs. We re-screened Pezizales mushrooms collected in northern Israel between 2020–2022 using molecular tools based on DNA sequences of partial large subunit rRNA (28S), internal transcribed spacer (ITS), and partial heat shock protein (*Hsp90*) regions, along with macro images of each freshly collected samples. Trophic mode was determined through available literature and δ^13^C and δ^15^N stable isotope analysis along with a quadratic discriminator analysis (QDA) model to predict trophic modes: 112 collections were positively identified with identification of 23 genera; 23 taxa were resolved to the species level, 11 to approximate species, and 15 to the genus level. *Helvella* was the most species-rich genus; 25 species and species approximations were newly reported for Israel. Further molecular phylogenetic studies are needed to resolve species identity of the Israeli Pezizales. Most Pezizales collections were determined by trophic mode studies to be ectomycorrhizal, with a few saprotrophs. The QDA model yielded several samples with undefined nutritional behavior or a different trophic mode than previously inferred, suggesting that more in-depth study is needed to understand their trophic ecology. This study improves knowledge regarding species diversity, ecology, and evolution of Israeli Pezizales.

## 1. Introduction

The order Pezizales (Pezizomycetes, Ascomycota) includes important cup and truffle fungi that are particularly diverse in temperate regions or at high elevations worldwide [1]. Recent studies have reported that this order includes approximately 23 families, 200 genera, and 2000 species [2]. Among the families are the well-known and economically important Helvellaceae, Tuberaceae, Pezizaceae, and Morchellaceae. Most species form disk-shaped ascomata and many fruit on the surface of forest soil, dead wood, and dung; however, some species grow underground (hypogeous) and are referred to as truffles [1]. The Pezizales asci typically release their spores through the opening of a terminal or eccentric lid, called an operculum, but this feature has been lost in most of the truffles. Soil-inhabiting species often fruit in habitats with a high soil pH and low organic matter content, including disturbed sites [3,4]. Pezizales fungi play important ecological roles in the environment, exhibiting several trophic strategies: ectomycorrhizal (ECM), coprotrophic, saprotrophic, endophytic, and myco- or plant-parasitic. This diversity has garnered much attention in the scientific community, resulting in many studies of Pezizales taxonomy and trophic behaviors to better understand their roles in ecosystems and to manage their presence in natural habitats and cultivated fields [5,6,7]. Due to their high trophic diversity, Pezizales are also studied to understand evolutionary transitions, including the transition between saprotrophic and ECM nutritional modes [8,9]. Recent studies using genomic comparisons have suggested that such evolutionary transitions are accompanied by a reduction in lignocellulose-degrading enzymes from litter- and wood-degrading saprotrophs to ECM fungi, the latter utilizing fresh carbon sources directly from symbiotic host plants [8,10,11]. In parallel, the adoption of molecular tools over the last decade has resulted in considerable reclassification and changes in fungal nomenclature [12,13]. It is not unusual for molecular phylogenetics to lead to new classifications for some taxa, and this approach has been useful in delimiting species and identifying cryptic species clusters (e.g., [14,15]). Progress has also been made in identifying the potential trophic state of fungi using the δ^13^C and δ^15^N isotopic signatures of the fruiting bodies, which are differentially accumulated in ECM and saprotrophic taxa [16,17,18]. δ^13^C and δ^15^N can provide useful insights into the trophic mode because different types of fungi have different primary carbon and nitrogen sources. Hobbie et al., 2001 [18] summarized the general pattern seen for δ^13^C content as: plants < mycorrhizal fungi < litter-decay fungi < wood-decay fungi and the pattern for δ^15^N content as: plants < saprotrophic fungi < mycorrhizal fungi. The use of the isotopic signature to gather additional data on the nutritional status of a fungal taxon relies on the discrimination of carbon and nitrogen sources used by fungi that employ different trophic modes; ECM species tend to have higher δ^15^N and lower δ^13^C values than the saprotrophic taxa due to their use of older nitrogen sources from the soil and fresher carbon sources directly from living plants [19]. Hobbie et al. [18] reported that ECM fungi are 3.5 ± 6‰ depleted in δ^13^C and 5.7 ± 0.4‰ enriched in δ^15^N compared to saprotrophic fungi. This is related to the different carbon sources for the two nutritional groups, with ECM fungi typically using freshly produced carbon sources from the host plant and mineral nitrogen from the soil (along with additional fractionation expected as the fungus provides nitrogen to the host plant). In contrast, saprotrophic fungi typically use older environmental organic matter, such as dead plant material (litter) and organic nitrogen sources [17,20,21]. Isotopic signatures can help determine fungal taxa's trophic status [16,17,18,22,23]. Isotopic tools also enable in-depth studies on the evolutionary transitions between saprotrophic and mycorrhizal trophic modes and may help to pinpoint cases in which trophic modes are complicated or poorly understood [24,25,26]. Moreover, Mayor et al. [27] used isotopic signature data of 813 fungi across 23 sites to establish a database enabling the prediction of trophic modes of other mushrooms with unknown ecological roles, which can further serve to study the ecological strategies of fungi.

Israel is a Mediterranean country in the geographical transition zone between African and European climates and is the convergence zone for four phytogeographical regions (Mediterranean, Irano–Turanian, Saharo–Sindian, and Sudanese–Dekkan enclaves) positioned along a major ecoclimatic gradient [28]. Consequently, Israel is suggested to have exceptional floral richness [29]. The high climatic and soil diversity is hypothesized to support numerous Pezizales, as well as other macrofungi. The ecology of wild mushrooms in Israel (including Pezizales cup fungi and truffles) has been scarcely studied, garnering little attention from the scientific community compared to other Mediterranean countries [30,31]. More recently, however, interest in Israeli mushrooms and their ecology has increased. The Israeli Pezizales were studied by Barseghyan and Wasser [32,33,34], who identified taxa mostly based on morphological features, while only *Morchella* species were subjected to molecular discrimination [33]. Most of the Pezizales in Israel were reported to be coprotrophs, wood saprotrophs, and humus saprotrophs, based mostly on visual observations of their fruiting habits [34]. Other recent studies of Israeli Pezizales have focused on important edible genera, such as *Terfezia* [35,36,37,38] and *Morchella* [39,40,41].

This work aimed to (1) increase knowledge of Israeli Pezizales diversity compared to previous documentation of Barseghyan and Wasser [34] in order to detect new species; and (2) assess the trophic mode of newly collected Pezizales as being either ECM or saprotrophic according to the available literature [7,42], as well as according to δ^13^C/^δ15^N isotopic signatures from their ascomata. The isotopic signature data further served to examine the ecological role of each collection using a quadratic discriminator analysis (QDA) model [27]. This could lead to identifying possible nutritional behavior transitions of species due to local conditions compared to other world regions.

## 2. Materials and Methods

### 2.1. Sample Collection and Processing

We obtained Pezizales ascomata in conjunction with citizen scientist collectors from different sites and habitats around the Galilee region in northern Israel during 2020–2022, mainly in springtime. This region has a temperate climate with hot, rainless summers and mild to cool, rainy winters and mostly Mediterranean forests dominated by oak (*Quercus* spp.), pine (*Pinus* spp.), bay laurel (*Laurus nobilis*), *Pistacia* spp., *Ceratonia siliqua*, and *Arbutus andrachne* trees.

Each specimen was photographed in its habitat prior to collection. Descriptions of nearby vegetation and the site (forest floor, limestone wall, valley bed, dry stream bank, dead branch, soil) were recorded for each sample (Appendix A). Data recorded on site included a photograph with the location and the nearby vegetation. All data were gathered using a Google questionnaire that included an option for uploading pictures. Ascomata were subsampled for molecular identification and determination of δ^13^C and δ^15^N isotopic signatures. For molecular identification, mushrooms were subsampled using a sterilized knife (Opinel, Avignon, France), placed in a sterile 5 mL Eppendorf tube, washed once with sterile cetyltrimethylammonium bromide (CTAB) lysis buffer [43], and then covered with CTAB and stored at −20 °C for further molecular identification. For isotopic signature determination, part of the same ascoma or adjacent ascomata from the same collection were placed in plain paper envelopes. Samples were transported in a cooler to the laboratory within 24 h, cleaned of debris, frozen at −20 °C, and further freeze-dried and processed, as described below. Clean subsamples of fungal tissue were shipped in CTAB to the Bonito laboratory (Michigan State University, East Lansing, MI, USA) or to the Smith laboratory (University of Florida, Gainesville, FL, USA) for further molecular analyses. As a quality control check to ensure that the sequences obtained represented the samples collected, species determination from DNA sequences was cross-checked with the photographs of each sample.

### 2.2. Molecular Methods

DNA was extracted using a modified CTAB extraction procedure [43]. Samples in CTAB were first examined under a stereomicroscope to select the cleanest portion of the tissue and, when necessary, further cleaned using sterilized forceps and razor blades. Selected cleaned samples were then placed in 500 µL of fresh CTAB. The specimens were crushed and briefly vortexed and incubated in a dry bath at 65 °C for 1 h. Samples were then centrifuged at 15,000 rpm for 2 min; the supernatant was decanted into a new tube, to which 500 µL chloroform was added. This mixture was incubated at room temperature (24 °C) for 1 h. Samples were again centrifuged at 15,000 rpm for 15 min, the supernatant was decanted into a new microcentrifuge tube, and the chloroform extraction was repeated. Then, 400 µL cold isopropyl alcohol was added to each tube of the supernatant and the tubes were stored overnight at −20 °C. Samples were then centrifuged at 15,000 rpm for 15 min; the supernatant was pipetted out and the pellet was retained. Precipitated DNA pellets were washed with 200 µL of cold 70% ethanol and centrifuged for 15 min at 15,000 rpm. The ethanol was decanted, and the pellet was dried inside the tube in a dry bath at 37 °C for 30 min. DNA pellets were reconstituted in 50 µL Tris–EDTA buffer (pH 8) in a dry bath at 56 °C for 30 min. Quality was assessed in a NanoDrop 2000 spectrophotometer (ThermoFisher Scientific, Waltham, MA, USA).

The internal transcribed spacer region ITS1–5.8S–ITS2 of the nuclear ribosomal DNA (ITS) was amplified with primers ITS1F [43] and ITS4 [44], or, in some cases, both ITS and partial large subunit rRNA (28S) were amplified with ITS5 and LR3 [45]. When necessary, the 28S was amplified separately from ITS with the primers LROR and LR5 [41]. For *Helvella* DNA, the heat shock protein (*Hsp90*) was amplified using primers *Hsp90*f and *Hsp90*r [46]. Successful PCR amplification was determined by electrophoresis in 1.5% agarose gels and staining the PCR product with SYBR Green I (Molecular Probes, Eugene, OR, USA). Amplicons were cleaned with Exonuclease I (EXO) and shrimp alkaline phosphatase (SAP) enzymes. Sanger sequencing was performed by Genewiz (South Plainfield, NJ, USA) or Eurofins Genomics (Louisville, KY, USA) on an ABI Prism 3730xl capillary sequencer using Big Dye v3.0 or v3.1 terminator chemistry Chromatograms were manually checked for quality, edited where necessary, and low-quality ends were trimmed in Geneious Prime 2024.0.7 [47]. Sequences of the ITS, 28S, and *Hsp90* regions of our samples were submitted to NCBI GenBank (Table 1).

### 2.3. Phylogenetic Analysis and Trophic State Estimation Based on Molecular Data

Samples were identified based on a combination of NCBI BLAST (https://www.ncbi.nlm.nih.gov/, accessed on 4 December 2024) and phylogenetic analysis of ITS, 28S, and *Hsp90* sequences, with further confirmation by morphological analyses of photos and light microscopy of specimens (Appendix A). In most cases, species determinations were based on significant support of phylogenetic clades from highly similar (≥97% identity similarity) sequences. First, we used our newly generated sequences as queries for BLAST searches to obtain approximate identification and similar sequences from NCBI. We added our new Israeli sequences and highly similar sequences from NCBI to ITS alignments from various lineages of Pezizales from Healy et al., 2022 [24]. The ITS region is the currently accepted barcode for fungi and has proven useful for the identification of Pezizales [13,24,48]; however, 28S is also useful for the phylogenetic placement of many Pezizales taxa (e.g., [14,15]). Moreover, because it can be difficult or impossible to sequence ITS from species of *Helvella*, the *Hsp90* was also utilized as an alternative phylogenetic marker [46]. To identify *Helvella* species, we incorporated our *Hsp90* sequences from this study into an alignment with *Hsp90* sequence data from representative sequences from the most recent molecularly based phylogenetic treatments of *Helvella* [46,49,50,51,52]. To reduce the tree length for presentation, we trimmed some of the branches that did not contain Israeli species within a given clade. Datasets for each gene region were assembled and aligned in MAFFT v 7.471 [53]. Alignments were manually improved in Geneious Prime 2024.0.7 (https://www.geneious.com, accessed on 4 December 2024) [47]. Maximum likelihood analyses were performed for each individual locus with RAxML-HPC2 v 8.2.12 [54] using the GTRCAT substitution model with 1000 bootstrap replicates. Bayesian inference (BI) was performed for each locus with MrBayes 3.2.7 [55]) using the best-fit model of substitution according to the corrected Akaike information criterion, estimated in jModelTest 2.1 [56]. For ITS and LSU, the GTR+I+G model was chosen from 88 possible models. For *Hsp90*, the K80+G model was chosen. Four independent runs were executed using a chain length of 20 million generations, a sampling frequency of 1000, and the first 25% of the samples were discarded as burn-in. The runs were terminated after stationarity had been reached (when the standard deviation of split frequency ≥ 0.01). The mixing behavior of the chains was evaluated in Tracer v1.7.2 [57] to ensure that coverage was adequate. The resulting best tree for each alignment was visualized in FigTree v 1.4.4 (http://tree.bio.ed.ac.uk/, accessed on 5 December 2024). Nodes for the ML and BI phylogeny were considered to be statistically supported when bootstrap values were ≥70% for ML and probabilities ≥0.95 for BI. All analyses were run on the Cyberinfrastructure for Phylogenetic Research Science Gateway (CIPRES) 3.3 [58].

Taxa identified to the species level are listed by their full Latin epithet; taxa identified only to the genus level are listed with genus followed by ‘sp.’; and taxa identified to an approximate species are designated by the genus name followed by ‘cf.’ and the approximate species name. The phylogenetic tree generated for Appendix B was prepared in Adobe Illustrator v. CS5.1 (San Jose, CA, USA).

Previous analysis suggests that ECM taxa generally fall into monophyletic groups and that reversion from ECM to saprotrophic mode is rare or impossible [42]. Accordingly, we used available literature to identify the putative trophic state of each identified sample via comparisons with the FungalTraits database [59].

### 2.4. Stable Isotope Analysis

We directly compared the trophic mode of Pezizales collections inferred from the literature with the trophic mode inferred from stable isotope analysis of our local Israeli specimens. Representative lyophilized specimens (n = 89) were crushed with a clean, surface-sterilized mortar and pestle, and then stored at −80 °C. Samples (20 mg) were sent on dry ice to Silvatech (INRAE, Paris, France). Carbon (δ^13^C) and nitrogen (δ^15^N) isotope contents were determined by placing tin capsules containing up to 10 mg of dry material in an elemental analyzer (vario ISOTOPE cube, Elementar, Langenselbold, Germany) coupled, via a gas box interface, to a continuous flow isotope ratio mass spectrometer (Isoprime100 IRMS, Elementar UK, Cheadle, UK) available at Silvatech. The samples were burned at 1025 °C in excess oxygen. Then, the nitrogen oxide was reduced using a quartz tube filled with copper at 650 °C. Carbon dioxide was trapped at 35 °C by an adsorption column, while nitrogen (N_2_) passed through the thermal conductivity detector. Next, carbon dioxide was released from the adsorption column at 225 °C. Elementary gases were analyzed and detected by isotope ratio mass spectrometry (Isoprime100 IRMS). Nitrogen and carbon contents were expressed as a percentage of dry matter. The δ^13^C and δ^15^N values were expressed as delta values in ‰ relative to the isotope ratio of the Vienna Pee Dee Belemnite (VPDB) standard and to atmospheric N_2_, respectively.

### 2.5. Data Recording

We created a table that includes the information for each sampled ascoma: species identification, family, sequence NCBI accession number, ID, trophic state, niche, nearby vegetation, location, collector, and comparison to an authoritative encyclopedia of Pezizales species in Israel [34]. Two columns represent the trophic mode: one according to the literature, and the other as predicted by the QDA model of the isotopic signature data (Appendix A). All known basionyms were also checked using Index Fungorum for potential synonyms of each species (https://www.indexfungorum.org/names/names.asp, (accessed 1 July 2024).

### 2.6. Data Analysis and Statistics

The results of the identified samples were compared to previous Israeli Pezizales reported by Barseghyan and Wasser (2013) [34] and reported in Table 1. ‘Yes’ indicates that a taxon was previously reported from Israel, ‘no’ indicates no previous report from Israel, and ‘maybe’ indicates samples from our dataset that were not identified to the species level but whose genus has been previously reported from Israel. QDA of δ^13^C and δ^15^N values was performed to predict the affiliation of all Israeli Pezizales samples to a dataset of known saprotrophic and ECM fungi from Mayor et al. (2009) [27]. A quadratic model was chosen over a linear one due to the unequal variances of δ^13^C and δ^15^N values used to train the model. The QDA model was calculated in RStudio (2023.06.2+561) with R version 4.3.1 (R Core Team, Vienna, Austria) with the QDA function in the MASS package [58]. QDA model predictions of the trophic group (ECM or saprotrophic) with posterior probability values below 0.8 were designated as ‘undetermined’.

## 3. Results

### 3.1. Molecular Identification

**Figure 1:**
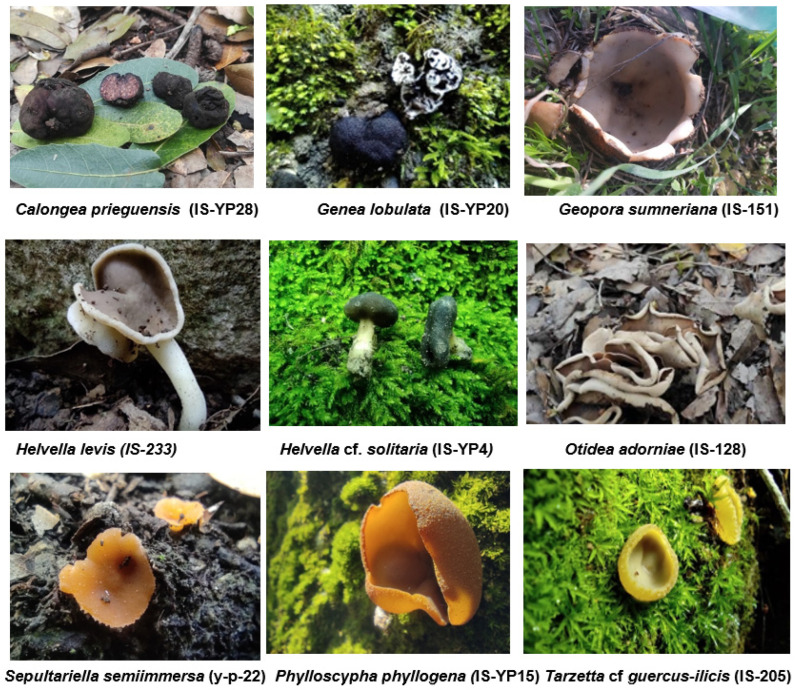
Newly recorded species identified in the present study. *Calongea* and *Genea* are new generic records for Israel. The other taxa represent new species records for Israel.

A total of 112 Pezizales collections were positively identified by DNA sequences of the ITS, 28S, and/or *Hsp90* regions and verified based on their morphology (Appendix A). Table 1 shows a condensed synopsis of Appendix A and provides information on one representative sample for each species. As summarized in Table 1, a total of 24 genera were recorded, with 50 different species identified. Among the identified species, 23 were definitively identified to the species level, whereas 11 others were identified to an approximate species, as designated by ‘cf.’ (e.g., *Helvella acetabulum* vs. *Helvella* cf. *acetabulum*), and 16 were identified only at the genus level. *Helvella* was the most commonly identified genus with 512 collections. Other dominant genera were (number of collections): *Paragalactinia* (12), *Dissingia* (5), *Peziza* (5), *Legaliana* (5), *Sarcoscypha* (5), and *Geopora* (4). The following genera were less common, with only one or two collections per genus: *Anthracobia*, *Calongea*, *Daleomyces*, *Elaiopezia*, *Galactinia*, *Genea*, *GeopyxisHumaria*, *Melastiza*, *Morchella*, *Otidea*, *Phaeopezia*, *Phylloscypha*, *Tarzetta*, *Scutellinia*, *Sepultariella*, and *Trichophaea*. Figure 1 provides images of selected newly recorded Israeli species. Most of the genera were found to belong to the families Pyronemataceae (12 genera) and Pezizaceae (9 genera). Only two genera were recorded in the Helvellaceae and Tarzettaceae, and only one genus each was recorded in Otideaceae, Morchellaceae, and Sarcoscyphaceae (Table 1, Appendix A)

After identifying and checking synonyms in Index Fungorum, we compared our documented species with those reported by Barseghyan and Wasser (2013) [34]. Of the 24 molecularly identified genera in the present work, the truffle genera *Genea* and *Calongea* had not been reported previously from Israel and are reported here for the first time (Table 1, Appendix A). At the species level, only 5 out of the 35 identified species or approximate species matched those presented by Barseghyan and Wasser (2013) [34] (i.e., *Dissingia (Helvella) leucomelaena*, *Helvella acetabulum*, *Paragalactinia succosa*, *Peziza varia*, *Sarcoscypha coccinea*). However, it is important to note that in some cases, taxa in Barseghyan and Wasser (2013) [34] were listed by an earlier synonym; therefore, all IDs were checked using Index Fungorum (https://www.indexfungorum.org/names/names.asp, accessed on 4 December 2024) for possible synonyms used by the preceding work. Out of the 23 fully identified species, 17 species are reported here for the first time: *Calongea prieguensis*, *Daleomyces bicolor*, *Genea lobulata*, *Geopora sumneriana*, *Helvella fuscolacunosa*, *Helvella inexpectata*, *Helvella lactea*, *Helvella levis*, *Helvella neopallescens*, *Helvella poculiformis*, *Helvella retinervis*, *Helvella solitaria*, *Otidea adorniae*, *Otidea bufonia*, *Phaeopezia apiculata*, *Phylloscypha phyllogena*, and *Sepultariella semiimmersa*. Another 10 taxa identified to the approximate species were also new records for Israel (Table 1, Appendix A). The approximate species and collections identified only to the genus level will require additional molecular and microscopic analysis for full and accurate identification. The species documented here but not previously reported by Barseghyan and Wasser (2013) [34] were further examined by online searches: only one additional species (*Geopyxis majalis*) was previously documented from Israel (Table 1).

### 3.2. Genus Helvella

*Helvella* was the most common genus among our Pezizales collections from northern Israel. For this genus, in addition to the ITS and 28S regions, the *Hsp90* locus was needed to further characterize the phylogenetic diversity [46]. The final alignment had 210 taxa and 275 positions. The tree topology from our phylogenetic analyses based on the *Hsp90* locus was consistent for the most part with the results from multi-locus analyses in recent revisions of this genus (Skrede et al., 2017 [46], Skrede et al., 2020 [49], Mao et al., 2023 [50]), but it lacked the robust support from those analyses. The four major clades identified by Mao et al. (2023) [50] were recovered, but the lacunosa clade was bisected. Species from three of these clades, (acetabulum, elastica, and lacunosa) contained representatives from northern Israel. No species from the crispa clade were collected, however. (Appendix B).

The phylogenetic trees generated from analyses of the *Hsp90* region or of the ITS region from 52 *Helvella* samples placed 34 of them within eight species (*Helvella acetabulum*, *H. fuscolacunosa*, *H. lactea*, *H. levis*, *H. neopallescens*, *H. poculiformis*, *H. retinervis*, and *H. solitaria*), and placed 13 of them close to six defined species (*Helvella* cf. *acetabulum*, *H*. cf. *calycina*, *H.* cf. *inexpectata*, *H*. cf. *poculiformis*, *H*. cf. *retinervis*, and *H*. cf. *solitaria*). Five additional taxa were only identified in the genus *Helvella* (Table 1, Appendix A, Appendix B). The following species were not recorded by Barseghyan and Wasser (2013) [34]: *Helvella fuscolacunosa*, *H.* cf. *inexpectata*, *H. lactea*, *H. levis*, *H. neopallescens*, *H. poculiformis*, *H. retinervis*, and *H. solitaria*.

### 3.3. Trophic Ecology and Isotopic Analysis

We examined published literature and databases and performed a QDA of stable isotopes to better understand the trophic ecology of our Pezizales collections. We first categorized each taxon as ECM or saprotrophic according to the FungalTrait database [59]. Of the 51 molecularly identified taxa representing the 116 Pezizales collections (Table 1, Appendix A), the number of ECM taxa (39 taxa representing 98 collections) was much higher than that of the saprotrophic taxa (12 taxa, representing 18 collections). The taxa designated as saprotrophic according to the literature were *Anthracobia* sp., *Daleomyces bicolor*, *Daleomyces* sp., *Elaiopezia* sp., *Geopyxis majalis*, *Melastiza* sp., *Morchella* sp., *Peziza varia*, *Peziza* sp. *sensu strico*, *Phaeopezia apiculata*, *Sarcoscypha coccinea*, and *Scutellinia* sp.

**Figure 2:**
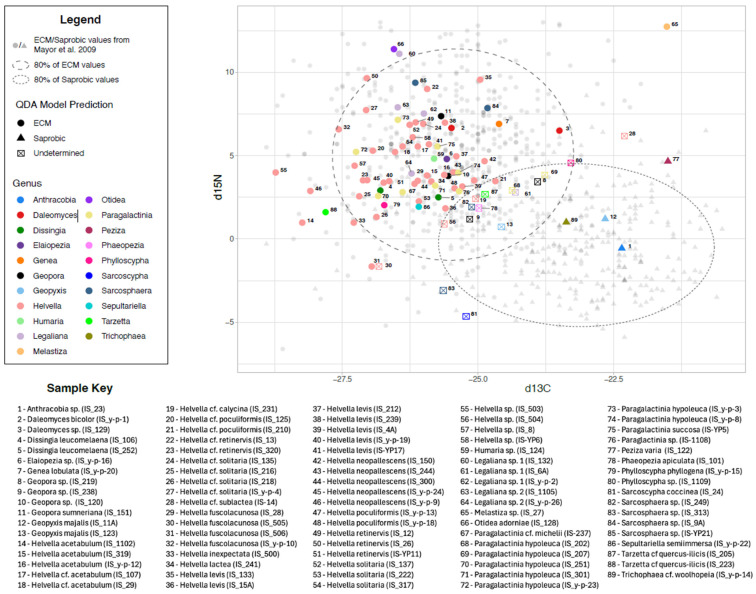
QDA model of predicted ectomycorrhizal (ECM) taxa (circles) and saprotrophic fungi (triangles) based on the dataset of known saprotrophic and ECM fungi with δ^13^C and δ^15^N values from Mayor et al. (2009) [27]. Predicted values from our dataset (colored shapes) are plotted against the background values from Mayor et al.’s (2009) [27] dataset (gray shapes). The figure shows the overall distribution and shape of the data used to make predictions. Values that were not predicted as either trophic mode with high confidence are indicated as ‘undetermined’ (open box with an X inside). Taxonomic determinations and specimen identification numbers are provided in the key because isotopic data were determined individually for each specimen.

We further examined stable δ^13^C and δ^15^N isotopes from the ascomata using QDA to predict trophic affiliation based on isotopic values of known ECM and saprotrophic fungi to provide additional insights into the trophic state of 89 representative collections (Figure 2). Of these, 9 were saprotrophic species according to the literature, and the other 80 were ECM species (Appendix A). Using the QDA model, only four collections belonging to four different species were confidently assigned as saprotrophic, whereas sixty-nine were confidently predicted as ECM, in accordance with the trophic mode obtained from the published literature. The putative saprotrophs *Daleomyces bicolor*, *Daleomyces* sp., *Elaiopezia* sp., and *Melastiza* sp. were determined as ECM according to the QDA model, whereas the ECM fungus *Trichophaea* cf. *woolhopeia* was predicted as saprotrophic by the model. An additional 17 samples were not confidently predicted by QDA as either saprotrophic or ECM taxa and were designated ‘undetermined’. These included *Geopora* sp., *Geopyxis majalis*, *Helvella* cf. *sublactea*, *Helvella fuscolacunosa*, *Legaliana* sp. 1, *Paragalactinia* cf. *hypoleuca*, *Phaeopezia apiculata*, *Phylloscypha* sp., *Sarcoscypha coccinea*, *Sarcosphaera* sp., and *Tarzetta* cf. *quercus-ilicis*.

**Figure 3:**
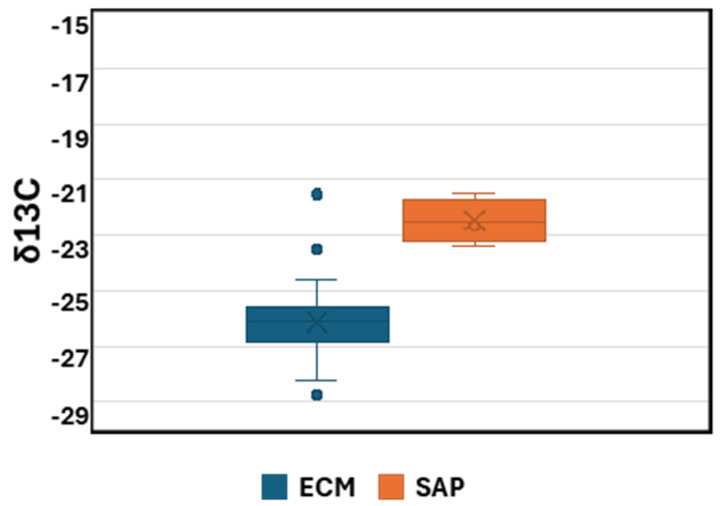
Boxplot of δ^13^C values for the ECM (n = 69) and saprotrophic (n = 4) ascomata defined according to the QDA model. Bars represent the minimum and maximum values. The points outside the lines are outliers that differ significantly from the rest of the data.

The samples predicted as ECM or saprotrophic according to the QDA model were clearly separated by their δ^13^C values, where saprotrophs had higher δ^13^C values (mean value of −22.4) than the ECM samples (mean −26.2) (Figure 3). The δ^15^N signature was less well defined for both ECM fungi and saprotrophs, being 11.3 to −3.1 and 12.7 to −4.6, respectively (Figure 2, Table 1).

## 4. Discussion

Pezizales are a large and diverse order of Fungi, with ca. 2000 species exhibiting a wide range of ecological functions in the environment, particularly in forests. They are found in diverse habitats worldwide, including in the Mediterranean Basin, such as Israel [30,32,33,34]. In an earlier morphological investigation of the Pezizales in Israel, Barseghyan and Wasser (2013) [34] reported finding 115 species, belonging to 37 genera and eight families. These taxa were classified into six trophic groups: saprotrophs, carbotrophs (post-fire saprotrophs), coprotrophs (dung saprotrophs), xylotrophs (wood saprotrophs), parasites, and ECM fungi. Most Pezizales species were reported as saprotrophs, coprotrophs, and carbotrophs. Here, we reassessed the Pezizales diversity in northern Israeli forests based on fresh collections of specimens obtained over a 2-year period in collaboration with citizen scientists. Collections were documented from different sites with diverse vegetation and soils, but they were all found in a relatively small region with a temperate climate.

### 4.1. Diversity of Pezizales in Israel

This is the first study to broadly apply both molecular and isotope analyses to Pezizales in Israel, resulting in a score of new Pezizales records for the country. Using molecular means, the current new screening of Pezizales mushrooms in northern Israel detected species not mentioned in the previous work of Barseghyan and Wasser [32,33,34]. Since the most recent work compiling records of Pezizales in Israel [34], there have been major revisions of some genera, most notably *Peziza* [60] and *Helvella* [46,49]. The considerable change in nomenclature and phylogenetic understanding over the last 10 years complicates direct comparisons between the morphologically based studies of Barseghyan and Wasser [32,33,34] and our molecular study.

To determine new species of Pezizales recorded in Israel, the names obtained in this work were checked against Index Fungorum to compare new names with old synonyms. This was performed because many of the fungi identified by Barseghyan and Wasser [34] have new taxonomic names reflecting an increasing awareness of the phylogenetic diversity within Pezizales. For example, *Calongea prieguensis* (Figure 1) was first described as *Pachyphloeus prieguensis* (=*Pachyphlodes prieguensis*) [61]. Healy et al., 2009 [62] used phylogenetic analysis to show that, despite morphological similarities, *C. prieguensis* is not closely related to *Pachyphlodes*. This species is relatively rare and was reported from only a few collections globally [62]. Its presence in Israel reflects a distribution of this species that is wider than previously realized.

*Calongea* is reported from Israel here for the first time, along with another truffle-forming genus, *Genea* (Figure 1). Neither of these genera was mentioned in the earlier comprehensive encyclopedia of operculate Pezizales of Israel [34]. In addition to the record of new genera, we found 16 additional species that had not been previously recorded. This relatively high proportion of new records of Pezizales species in Israel could be because our specimens were collected from northern Israel, which has been under-sampled compared to other parts of the country. In addition, molecular phylogenetics may have revealed taxonomic results that differ from those of the identification based on morphological characteristics alone. Furthermore, some species may be cryptic, with similar morphology despite being genetically distinct. Such variation supports the need for molecular identification to confirm species' identities. Another issue is that field collections may include non-target micro-fungi, such as mycoparasites, that might confound molecular analyses. In a few cases, we were not able to generate useful sequence data. The use of an axenic cultures could overcome problems of contamination, but it is difficult to obtain single-spore cultures from field collections, where sterile work is impossible. Sow et al., 2004 [63] demonstrated an approach for single-spore culture that successfully produces clean cultures of field-collected samples while preserving the specimen. This approach could be considered for future studies.

In our study, *Helvella* had the highest incidence of reported collections. *Helvella* species in Israel have been extensively studied [32,33,34], using macroscopic and microscopic observations for identification with classification according to Dissing (1966) [64]. Interestingly, all *Helvella* species reported by those authors differed from what we found in the present work, except for *H. acetabulum*. Other *Helvella* species found in their work but not identified in the present work include *H. atra*, *H. chinensis*, *H. crispa*, *H. elastica*, *H. ephippium*, *H. lacunosa*, *H. pezizoides*, *H. phlebophora*, *H. queletii*, and *H. spadicea*. The wide differences between the *Helvella* species classified in the present vs. earlier work highlight the importance of using molecular data rather than relying on phenotypic characterizations alone. Based on our phylogenetic analysis of *Hsp90*, ITS, and 28S DNA sequences, all of the species found in the current study are also found in Europe and the Mediterranean basin.

Our collections of Pezizales from Israel belong mainly to the Pyronemataceae and Pezizaceae, along with two genera in Helvellaceae and Tarzettaceae and one genus for each of the families Otideaceae, Morchellaceae, and Sarcoscyphaceae. In their comprehensive summary of the Israeli Pezizales, Barseghyan and Wasser (2013) [34] reported nine families: Ascobolaceae, Ascodesmidaceae, Helvellaceae, Morchellaceae, Pezizaceae, Pyronemataceae, Sarcoscyphaceae, Sarcosomataceae, and Tuberaceae. Globally, most Pezizales families are distributed in temperate zones and include the Helvellaceae, Morchellaceae, Pezizaceae, Rhizinaceae, and many of the Pyronemataceae. Several other families are more abundant in tropical or subtropical regions, including Sarcoscyphaceae, Sarcosomataceae, and Wynneaceae [65]. Given the limited geographical reach of the study (mostly Galilee and lower Carmel regions), broader screening efforts are likely to show even higher diversity and more varied ecological roles than previously described for Pezizales in Israel. For example, *Morchella* species are abundant in Israel [33,39,41], yet only one sample was found in the present screening work.

### 4.2. Trophic Ecology of Pezizales in Israel

The revision of *Peziza sensu lato* has helped clarify the trophic ecology of Pezizales and has provided additional evidence for the tendency towards the conservation of fungal trophic modes (in particular ECM) at the genus level [2,42]. There has long been confusion surrounding the genus “*Peziza*”, the cup-shaped species from which the class derives its name. Historically, this genus had a much broader concept than it does today and included lineages of unrelated species from multiple families and classes. The type species is the saprobic *Peziza vesiculosa*. Hansen et al. (2002, 2005) [66,67] used a multi-locus analysis to delimit the monophyletic clade that includes the type species *P. vesiculosa*. This is the currently accepted delimitation of *Peziza*, and these species are referred to as *Peziza sensu stricto*. All members of the genus *Peziza* are known to be saprobic and/or endophytic/endolichenic [26,65,66,67]. Since then, molecular phylogenetics have helped to sort out species of *Peziza sensu lato* (species that are not in a monophyletic lineage with *P. vesiculosa*). For example, Van Vooren (2020) [60] recently transferred several *Peziza sensu lato* species to *Daleomyces, Elaiopezia*, *Geoscypha*, *Ionopezia*, *Legaliana*, *Malvipezia, Paragalactinia*, *Phaeopezia*, and *Phylloscypha.* The species in *Legaliana* and *Paragalactinia* were determined to be ECM based on a combination of molecular sampling of ECM roots, isotopic data, and phylogenetic analyses [7,42]. However, a few species remain to be transferred, which can continue to cause some confusion. For example, among the Israeli species, *Peziza* cf. *azureoides* should be transferred to a different genus. Van Vooren (2020) [60] suggested *Galactinia* as the appropriate genus for the small clade of fungi to which the Israeli collection (IS_7) belongs, but an official transfer was deferred until additional data and analyses become available. Therefore, here we refer to these misclassified species as *Peziza sensu lato*.

From a nutritional point of view, most of the Pezizales have been traditionally considered to be saprotrophs [2]. Accordingly, most of the Israeli Pezizales were reported as saprotrophs by Barseghyan and Wasser (2013) [34]. However, most of the Pezizales collected in the present work were identified as ECM based on the available literature. Most of our collections were found on the forest floor under oak trees or in bryophyte-enriched habitats, including among bryophyte colonies. The over-representation of ECM taxa in our collections may reflect the high diversity of the Pezizales in Israel and might also be related to the specific geographical area and habitats in northern Israel, where many of our collections were found. The discrepancy between the historical literature and our data may also be due to the fact that some ECM taxa can occur on burned soil or appear to be fruiting on well-decayed wood but may still obtain most of their carbon from living plant symbionts [42,68]. For example, the previous classification of taxa as saprotrophs of humus might have relied too heavily on the habitat rather than the actual carbon source. This discrepancy could also arise from unique species identification of the same morphotype due to the molecular work compared to previous phenotypic identification work, resulting in different related trophic modes. Also, it could be a result of the different habitats and geographical zones, as well as seasonality. Wider screening over other areas and habitats in Israel might identify more saprotrophs.

Focusing on taxa that were well represented in our dataset (species with three or more samples), we found that there was a weak correlation of δ^13^C values, i.e., there were variations in δ^13^C level in different ascomata of the same species found in different locations (Figure 2). Additional sampling and isotope analysis are needed to further explore the extent of within-species and within-genus isotopic variation. Hobbie and Agerer (2010) [69] suggested that the diversity of δ^13^C levels in the ascomata of different species might be because carbon allocation to mycorrhizal fungi correlates with fungal strategies of growth, colonization, and exploration. The nearly ubiquitous presence of bryophytes might also be a contributing factor in this regard, given that Pezizales are known to also live as endophytes in mosses [24].

As for δ^15^N accumulation, in the present work, samples from species that were considered ECM and saprotrophic according to the literature accumulated a wide range of δ^15^N (from 12 to −4.6). This is in contrast to Hobbie et al. (1999) [19], who reported that ECM species tend to have higher δ^15^N and lower δ^13^C values than saprotrophic species, which could be related to the differences among species' abilities to access different forms of organic nitrogen sources in the soil, as well as the different soil contents of δ^15^N. For example, many soils are enriched with fresh nitrogen by nitrogen-fixing bacteria with a low δ^15^N, especially in bryophyte zones [70]. Another explanation, suggested by Hobbie and Agerer (2010) [69], is that, like δ^13^C accumulation in the ascoma, fungal δ^15^N reflects fungal exploration strategies and hyphal properties (i.e., fungi that are high-biomass ECM ‘exploration types’ have 4–7‰ more enriched ^15^N than fungi that are low-biomass ECM ‘exploration types’). Similar to their saprotrophic ancestors, ECM fungi also inhabit both soil and plant root niches, and they have access to mineral and organic nutrients in the soil layers. Hence, they can acquire a large array of macronutrients, including inorganic and organic nitrogen compounds and inorganic and organic phosphate compounds that are not bioavailable to plants [8]; each of these nutrient pools may impact the resulting isotopic signature in the fruiting bodies.

Our use of the QDA model to predict ECM and saprotrophic fungi based on a dataset of known ECM and saprotrophic fungal δ^13^C and δ^15^N values [27] revealed that most of the isotopic levels of the Israeli samples were congruent with the trophic state suggested by the FungalTraits database. However, there were several exceptions. For example, *Daleomyces bicolor*, *Daleomyces* sp., *Elaiopezia* sp., and *Melastiza* sp. were designated saprotrophic based on previous studies and the FungalTrait database but were determined to be ECM by the QDA model. This could be related to these samples’ low δ^13^C and high δ^15^N values, running counter to Hobbie et al.’s (1999) [19] reported that ECM species have higher δ^15^N and lower δ^13^C values than saprotrophic taxa; *Daleomyces bicolor* and *Elaiopezia* sp. had values of 6.65 and 4.79 for δ^15^N and −25.65 and −25.58 for δ^13^C, respectively, placing them in the ECM group. Many terricolous fungi have been reported to have low δ^13^C and high δ^15^N values [71]. On the other hand, *Trichophaea* cf. *woolhopeia* was considered to be ECM based on previous sequences from ECM roots and data from the FungalTraits database, but this taxon was defined as saprotrophic by the QDA model. In addition to these taxa, whose isotopic signatures conflicted with their suspected nutrition-acquiring mode based on the FungalTrait database, we also found an additional 17 samples that were ‘undetermined’ based on low support for their placement as either ECM or saprotrophic. This might be explained by the high variability of local available nutritional elements, resulting in high variability of the isotopic levels obtained in different ascomata of the same species. More in-depth work is required to study the unexpected results and ‘undetermined’ samples in order to learn more about their trophic ecology. Approaches that could be employed include analyzing isotopes from additional samples and the related environment (to determine the variation in isotopic signatures across species and habitats), in vitro culturing and synthesis experiments with plants, and more complete genomic assessment of their carbohydrate-active enzymes (CAZymes). Potential differences in fungal nutrient sources may also help explain observed inconsistencies in QDA predictions vs. recorded trophic modes of some Pezizales samples. Fungi living on litter are usually depleted in ^13^C relative to wood-decay fungi but enriched in ^13^C relative to ECM fungi [18]. For example, the saprotroph *Sarcoscypha coccinea* is a wood-decay fungus [72] but had a δ^13^C value below −25, similar to the ECM samples, whereas *Geopyxis majalis* is considered a saprotroph and endophyte, and also has a δ^13^C value of less than −24 [73,74]. ECM-forming Pezizales species with exceptionally high δ^13^C values might also be opportunistic saprotrophs, but there were too few samples of each of the species in the ‘undetermined’ group of fungi to draw any definite conclusions regarding their trophic state. Due to their close phylogenetic relationships with ECM taxa and shared niche in the upper soil layers, litter-decay fungi often overlap isotopically (for both nitrogen and carbon) with ECM fungi [11,18]. It could, therefore, be important to determine the substrate’s stable isotopes as well, because recently deceased wood could have an isotopic signature similar to that of live wood. While isotopic data provide a useful tool for examining trophic modes, our results indicate that isotopic values and predictive analyses, such as QDA, cannot unequivocally determine trophic status. Notably, in the dataset used to train the QDA model, there was a visible overlap between the δ^13^C and δ^15^N values of some known ECM and saprotrophic fungi (Figure 2), suggesting that there is not always a clear demarcation between ECM and saprotrophic fungi’s δ^13^C and δ^15^N profiles, even if general trends exist. Furthermore, a whole-genome analysis might provide insights into their gene repertoire and metabolic potential [8,75]. Although ECM taxa have retained some saprotrophic capabilities and extracellular enzymatic activities, there is generally a reduction in CAZymes with an increase in transposable elements and small secreted proteins during the transition from saprotrophic to ECM nutrition [10,76].

## 5. Conclusions

Molecular identification of freshly collected mushroom species of the order Pezizales from a range of habitats in northern Israel resulted in a high proportion of newly recorded species compared to previous studies that relied solely on morphological analyses [30]. Some of the discrepancies between our results and previous work may be due to changes in fungal nomenclature resulting from modern phylogenetic sequence-based fungal classification, and the higher resolution provided by this approach. In terms of their trophic ecology, most of the samples were found to be ECM, in contrast to previous studies in which most of the samples were designated as saprotrophs. That could be related to the different habitats screened, different pinpoint coordinates of previous specimen sampling, climate change, and seasonality. This underscores the importance of conserving old forests in this region because the ECM relationships are often highly specific and dependent on plant hosts [77]. Using δ^13^C and δ^15^N stable isotope analysis on our Israeli specimens to discriminate between ECM and saprotrophic fungi yielded interesting results and unexpected discrepancies. As molecular technology evolves from barcoding and phylogenetics for species identification to whole-genome analysis, it will be interesting to revisit the genomic capacity of some of these fungi. Whole-genome data could provide more information on the enzymes involved in the nutritional behavior of each fungus sampled at each site, and perhaps more precisely define the trophic mode of Pezizales and other fungi.

## Figures and Tables

**Table 1:** Summary of Pezizales species, family, NCBI accession numbers of DNA sequences used to identify the specimen, ID code of a representative sample for each taxon, trophic mode according to the literature, and whether these taxa were reported previously by Barseghyan and Wasser (2013) [34]. Data for the full list of samples of all species are presented in Appendix A (where trophic mode is defined according to both the literature and the quadratic discriminator analysis [QDA] model of the isotopic signature).

Species	Family	GB Accession no	Sample ID	Reported by Barseghyan and Wasser [34]
a. Ectomycorrhizal species				
*Calongea prieguensis*	Pezizaceae	OR142392 (ITS)	IS_YP28	No
*Dissingia* cf. *leucomelaena*	Helvellaceae	OR142369 (ITS); OR141932 28S	IS_316	Maybe
*Dissingia leucomelaena*	Helvellaceae	OR141907(28S); PQ072244 (*Hsp90*)	IS_102	Yes
*Genea lobulata*	Pyronemataceae	OR142387 (ITS)	IS_YP20	No
*Geopora* sp.	Pyronemataceae	OR142351(ITS)OR141925 (28S)	IS_219	Maybe
*Geopora sumneriana*	Pyronemataceae	OR142344 (ITS)	IS_151	No
*Helvella acetabulum*	Helvellaceae	OR142373 (ITS)	IS-1102	Yes
*Helvella* cf. *acetabulum*	Helvellaceae	OR141909(28S); PQ072246 (*Hsp90*)	IS_107	Yes
*Helvella* cf. *calycina*	Helvellaceae	OR142353 (ITS)	IS_231	No
*Helvella* cf. *inexpectata*	Helvellaceae	OR142370 (ITS) PQ072271 (*Hsp90*)	IS_500	No
*Helvella* cf. *poculiformis*	Helvellaceae	OR141910(28S); PQ072247 (*Hsp90*)	IS_125	No
*Helvella* cf. *retinervis*	Helvellaceae	OR141895(28S); PQ072239 (*Hsp90*)	IS_13	No
*Helvella* cf. *solitaria*	Helvellaceae	OR142350(ITS); OR141924(28S); PQ072260 (*Hsp90*)	IS_218	No
*Helvella fuscolacunosa*	Helvellaceae	OR142371(ITS); PQ072272 (*Hsp90*)	IS_YP10	No
*Helvella lactea*	Helvellaceae	OR142347(ITS); PQ072253 (*Hsp90*)	IS_203	No
*Helvella levis*	Helvellaceae	OR142359(ITS); OR141929(28S); PQ072263 (*Hsp90*)	IS_239	No
*Helvella neopallescens*	Helvellaceae	OR142362(ITS); OR141930(28S); PQ072265 (*Hsp90*)	IS_244	No
*Helvella poculiformis*	Helvellaceae	OR141922(28S); PQ072258 (*Hsp90*)	IS_213	No
*Helvella retinervis*	Helvellaceae	OR141899(28S); PQ072241 (*Hsp90*)	IS_26	No
*Helvella solitaria*	Helvellaceae	OR141926(28S); PQ072261 (*Hsp90*)	IS_222	No
*Helvella* sp.	Helvellaceae	PQ072281 (*Hsp90*)	IS_503	Maybe
*Humaria* sp.	Pyronemataceae	OR142340 (ITS)	IS_124	Maybe
*Legaliana* sp. *1*	Pezizaceae	OR142343(ITS); OR141912 (28S)	IS_132	Maybe
*Legaliana* sp. *2*	Pezizaceae	OR142391 (ITS); OR141937 28S	IS_YP26	Maybe
*Otidea adorniae*	*Otideaceae*	OR142341 (ITS)	IS_128	No
*Otidea bufonia*	*Otideaceae*	OR142393 (ITS)	IS_YP35	No
*Paragalactinia* cf. *michelii*	Pezizaceae	OR142357 (ITS)	IS_237	Yes
*Paragalactinia* cf. *hypoleuca*	Pezizaceae	OR142349(ITS); OR141918 (28S)	IS_207	No
*Paragalactinia succosa*	Pezizaceae	OR141933 (2S)	IS_YP5	Yes
*Paragalactinia* sp.	Pezizaceae	OR141928 (28S)	IS_224	Maybe
*Peziza* cf. *azureoides*	Pezizaceae	OR142318 (ITS)	IS_7	No
*Phylloscypha phyllogena*	Pezizaceae	OR142384 (ITS)	IS_YP15	No
*Phylloscypha* sp.	Pezizaceae	PP974221 (ITS)	IS_1109	Maybe
*Sarcosphaera* sp.	Pezizaceae	OR142319 (ITS)	IS_9	Maybe
*Sepultariella semiimmersa*	Pyronemataceae	OR142388 (ITS)	IS_YP22	No
*Tarzetta* cf. *quercus-ilicis*	Tarzettaceae	OR142352(ITS); OR141927 (28S)	IS_223	No
*Trichophaea* cf. *woolhopeia*	Pyronemataceae	OR142383 (ITS)	IS_YP14	No
b. Saprotrophic species				
*Anthracobia* sp.	Pyronemataceae	OR142326 (ITS)	IS_23	Maybe
*Elaiopezia* sp.	Pezizaceae	OR142385(ITS); OR141935 (28S)	IS_YP16	Maybe
*Daleomyces* sp.	Pezizaceae	OR142342 (ITS)	IS_129	Maybe
*Daleomyces bicolor*	Pezizaceae	OR142376 (ITS)	IS_YP1	No
*Geopyxis majalis*	Tarzettaceae	OR142339 (ITS)	IS_123	No *
*Melastiza* sp.	Pyronemataceae	OR142329(ITS); OR141900 (28S)	IS_27	Maybe
*Morchella* sp.	Morchellaceae	OR142322 (ITS)	IS_17A	Maybe
*Peziza varia*	Pezizaceae	OR142338 (ITS)	IS_122	Yes
*Peziza* sp. *sensu stricto*	Pezizaceae	OR142324(ITS); OR141897 (28S)	IS_21	Maybe
*Phaeopezia apiculata*	Pezizaceae	OR142334(ITS); OR141906 (28S)	IS_101	No
*Sarcoscypha coccinea*	Sarcoscyphaceae	OR142330(ITS); OR141902 (28S)	IS_30	Yes
*Scutellinia* sp.	Pyronemataceae	OR142337 (ITS)	IS_121	Maybe

* This species was mentioned recently in another publication: https://mushrooms.org.il/item/330 (accessed on 20 May 2025).

**Figure A1:**
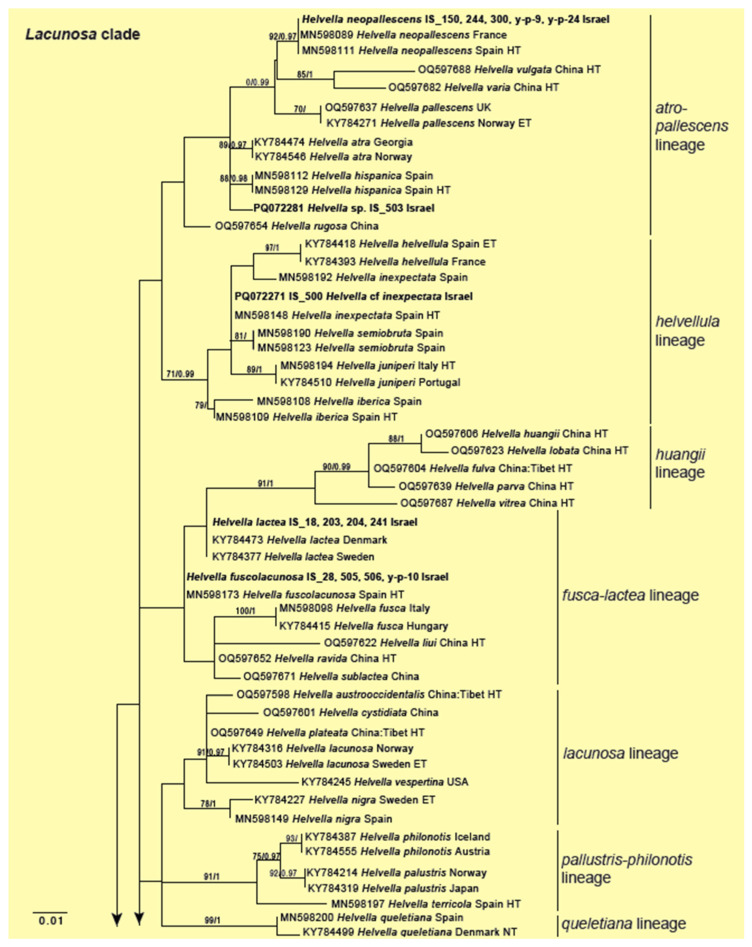

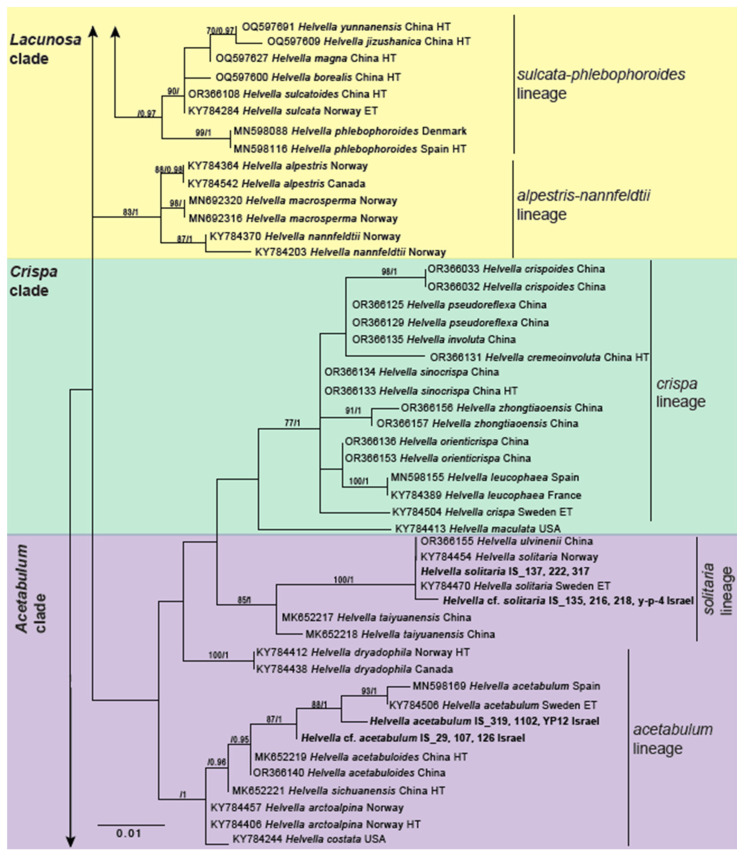

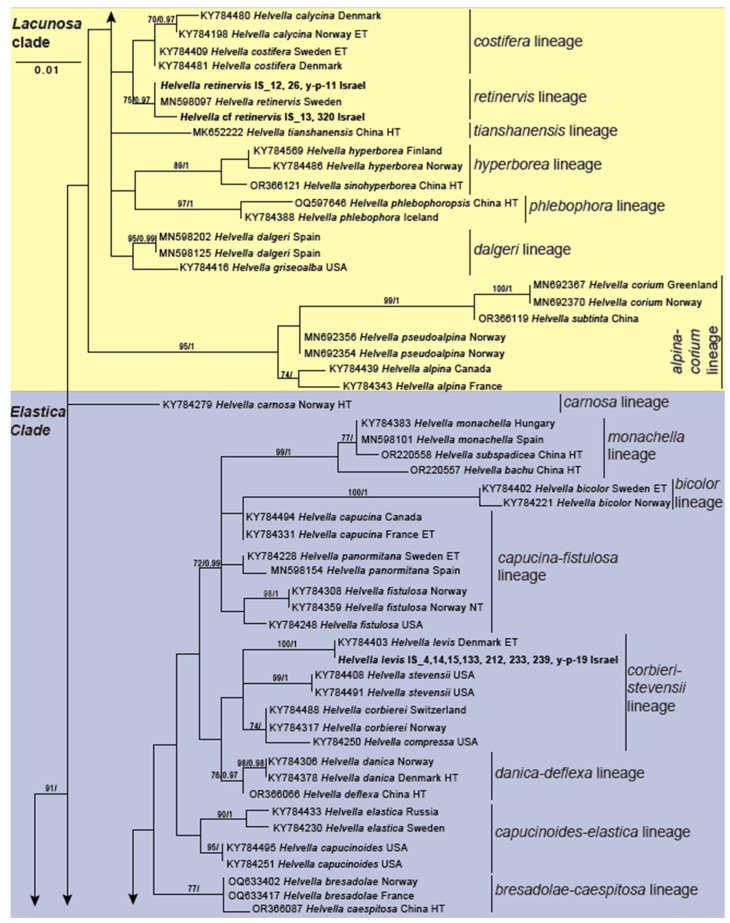

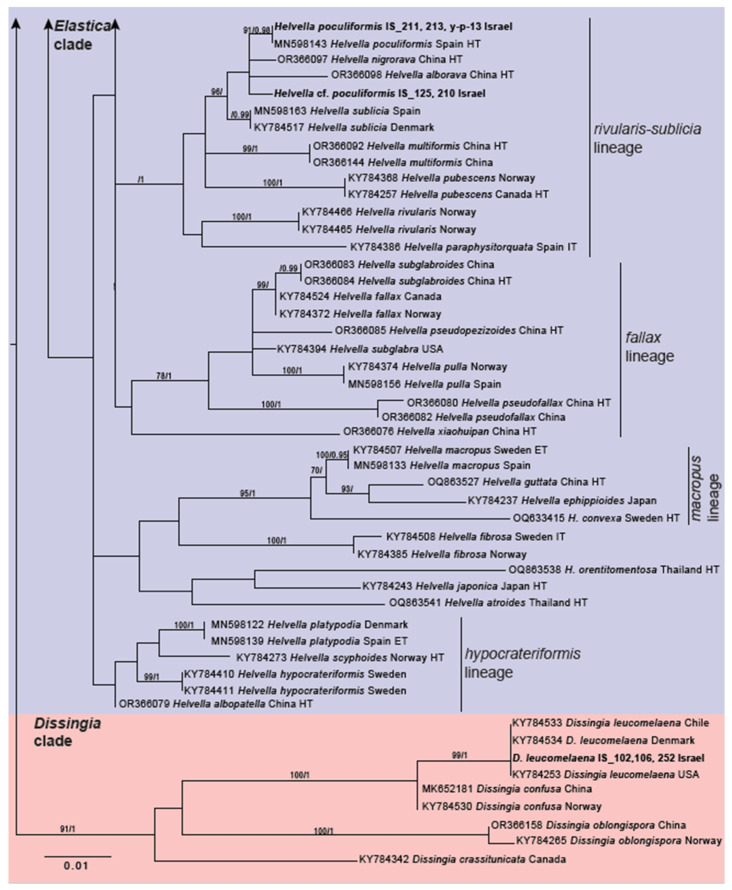
Phylogenetic tree of Israeli *Helvella* species based on the *Hsp90* region (four pages). Sequences from the Masaphy collections are in bold text. Bootstrap support values ≥ 70% are shown on the left and posterior probabilities ≥0.95 are shown on the right of the hash mark at branch nodes. Israeli species are indicated in bold, and terminals include all samples with identical sequences. The tree was midpoint rooted. Terminals include currently accepted names along with type designations. HT = holotype, IT = isotype, ET = epitype, NT = neotype. Colors correspond with major clade designations, indicated to the upper left of each color block, following Mao et al., 2023 [50].

## Data Availability

The original contributions presented in this study are included in the article. Further inquiries can be directed to the corresponding author.

## Appendix A

**Table A1:** Complete list of identified samples: species, family, GenBank accession numbers of the partial sequence of large-subunit rRNA 28S, internal transcribed spacer (ITS), and heat shock protein (*Hsp90*) regions of the DNA, species identity, habitat, nearby vegetation, site, collector, date of collection, trophic mode (according to the literature and according to the quadratic discriminator analysis [QDA] model), and whether the species was previously reported in Barseghyan and Wasser [34]. EO—Ezra Orlofsky; YS—Yaniv Segal; SM—Segula Masaphy; CC—community contribution. The ‘Trophic mode according to QDA model’ column includes only the samples that were analyzed for isotopic signature. Samples that were not analyzed are marked as (-).

Species	Family	ITS GB Accession Number	ID	Habitat, Nearby Vegetation, Collected by and Date of Collection Data	Trophic Mode According to Literature Versus According QDA Model	Reported by Barseghyan and Wasser 2013?
*Calongea prieguensis*	Pezizaceae	OR142392 (ITS)	IS_y-p-28	Forest floor; oak, bay; West Galilee Region, YS, 30 March 2022	ECM/-	No
*Dissingia* cf. *leucomelaena*	Helvellaceae	OR142368 (ITS)	IS_315	Forest floor; pine, bryophyte, grass, orchid; Merom Ha-Galil Region, EO , 15 March 2021	ECM/-	Maybe
*Dissingia* cf. *leucomelaena*	Helvellaceae	OR142369 (ITS); OR141932 (28S)	IS_316	Forest floor; pine, bryophytes, grass, orchid; Merom Ha-Galil Region, EO, 15 March 2021	ECM/-	Maybe
*Dissingia leucomelaena*	Helvellaceae	PQ072266	IS_252	Forest floor; cut pine, bryophytes; grass and herbs, Merom Ha-Galil Region, EO, 15 March 2021	ECM/ECM	Yes
*Dissingia leucomelaena*	Helvellaceae	PQ072245	IS_106	Trail side; bryophytes, oaks, asparagus, cyclamens, weeds; Naftali Mountains Region, EO, 07 February 2021	ECM/ECM	Yes
*Dissingia leucomelaena*	Helvellaceae	OR141907 (28S); PQ072244 (*Hsp90*)	IS_102	Trail side; bryophytes, oaks, asparagus, cyclamens, weeds; Naftali Mountains Region, EO, 07 February 2021	ECM/ECM	Yes
*Genea lobulata*	Pyronemataceae	OR142387 (ITS)	IS_y-p-20	Forest floor; under pine tree, bryophytes; West Galilee Region, YS, 15 March 2022	ECM/ECM	No
*Geopora* sp. 1	Pyronemataceae	OR142351 (ITS); OR141925 (28S)	IS_219	Forest floor; bryophyte, bay, oak; West Galilee Region, EO and YS, 21 February 2021	ECM/Undetermined	Maybe
*Geopora* sp. 1	Pyronemataceae	OR142358 (ITS)	IS_238	On limestone wall; bryophyte; West Galilee Region, YS, 14 March 2021	ECM/Undetermined	Maybe
*Geopora* sp. 1	Pyronemataceae	OR142336 (ITS)	IS_120	Forest floor; oak, bryophyte; West Galilee Region, EO and YS, 16 March 2021	ECM/ECM	Maybe
*Geopora sumneriana*	Pyronemataceae	OR142344 (ITS)	IS_151	Forest floor; oak, pine; Golan Heights Region, EO and IS, 07 March 2021	ECM/ECM	No
*Helvella acetabulum*	Helvellaceae	PQ072269 (*Hsp90*)	IS_319	Forest floor; oak; West Galilee Region, EO and YS, 16 March 2021	ECM/ECM	Yes
*Helvella acetabulum*	Helvellaceae	OR142373 (ITS)	IS_1102	Forest floor; oak; West Galilee Region, YS, 15 March 2022	ECM/ECM	Yes
*Helvella acetabulum*	Helvellaceae	PQ072277 (*Hsp90*)	IS_y-p-12	Valley bank; bryophytes; West Galilee Region, YS, 14 March 2022	ECM/ECM	Yes
*Helvella* cf. *acetabulum*	Helvellaceae	PQ072243 (*Hsp90*)	IS_29	Side of trail; *Rhamnus* sp, *Cercis siliquastrum,* oak, asparagus, *Retama* sp, bryophytes; Naftali Mountains Region, EO, 26 January 2021	ECM/ECM	Yes
*Helvella* cf. *acetabulum*	Helvellaceae	OR141909 (28S); PQ072246 (*Hsp90*)	IS_107	On slope near trail; *Rhamnus* sp, oak, bryophyte; Naftali Mountains Region, EO, 14 February 202	ECM/ECM	Yes
*Helvella* cf. *acetabulum*	Helvellaceae	OR141911 (28S); PQ072248 (*Hsp90*)	IS_126	Forest floor; bay, oak; West Galilee Region, EO and YS, 16 March 2021	ECM/-	Yes
*Helvella* cf. *calycina*	Helvellaceae	OR142353 (ITS)	IS_231	Forest floor; oak; West Galilee Region, EO and YS, 01 March 2021	ECM/Undetermined	No
*Helvella* cf. *inexpectata*	Helvellaceae	OR142370 (ITS); PQ072271 (*Hsp90*)	IS_500	Forest floor; bryophytes, oak, pine, weeds; West Galilee Region, EO and YS, 11 February 2021	ECM/ECM	No
*Helvella* cf. *poculiformis*	Helvellaceae	PQ072255 (*Hsp90*)	IS_210	Forest floor; oak; Emek Yizrael Region, YS, 18 February 2021	ECM/ECM	No
*Helvella* cf. *poculiformis*	Helvellaceae	OR141910 (28S); PQ072247 (*Hsp90*)	IS_125	Forest floor rich soil; bay, oak; West Galilee Region, EO and YS, 16 March 2021	ECM/ECM	No
*Helvella* cf. *retinervis*	Helvellaceae	PQ072270 (*Hsp90*)	IS_320	Forest floor; oak; West Galilee Region, EO and YS, 16 March 2021	ECM/ECM	No
*Helvella* cf. *retinervis*	Helvellaceae	OR141895 (28S); PQ072239 (*Hsp90*)	IS_13	Forest floor; oak; West Galilee Region, EO and YS, 16 March 2021	ECM/ECM	No
*Helvella* cf. *solitaria*	Helvellaceae	OR141923 (28S); PQ072259 (*Hsp90*)	IS_216	Forest floor; oak, bay; West Galilee Region, EO and YS, 22 February 2021	ECM/ECM	No
*Helvella* cf. *solitaria*	Helvellaceae	OR142350 (ITS); OR141924 (28S); PQ072260 (*Hsp90*)	IS_218	Forest floor; oak, bay; West Galilee Region, EO and YS, 22 February 2021	ECM/ECM	No
*Helvella* cf. *solitaria*	Helvellaceae	PQ072273 (*Hsp90*)	IS_y-p-4	Valley floor; bryophytes; West Galilee Region, YS, 13 March 2022	ECM/ECM	No
*Helvella* cf. *solitaria*	Helvellaceae	OR141914 (28S); PQ072250 (*Hsp90*)	IS_135	Valley bed; bryophyte; West Galilee Region, EO and YS, 16 March 2021	ECM/ECM	No
*Helvella fuscolacunosa*	Helvellaceae	OR142371 (ITS); PQ072272 (*Hsp90*)	IS_505	Forest floor; oak, bay; West Galilee Region, EO and YS, 11 February 2021	ECM/Undetermined	No
*Helvella fuscolacunosa*	Helvellaceae	OR141901 (28S); PQ072242 (*Hsp90*)	IS_28	On side of trail; *Rhamnus* sp, *Cercis siliquastrum*, oak, asparagus, *Retama* sp, bryophytes; Naftali Mountains Region, EO and YS, 26 January 2021	ECM/ECM	No
*Helvella fuscolacunosa*	Helvellaceae	OR142372 (ITS)	IS_506	Forest floor; oak, bay; West Galilee Region, EO and YS, 11 February 2021	ECM/ECM	No
*Helvella fuscolacunosa*	Helvellaceae	OR142381 (ITS); PQ072275 (*Hsp90*)	IS_y-p-10	Valley bank; bryophytes, oak; West Galilee Region YS, 14 March 2022	ECM/ECM	No
*Helvella lactea*	Helvellaceae	OR142347 (ITS); PQ072253 (*Hsp90*)	IS_203	Forest floor; oak; West Galilee Region, EO and YS, 29 March 2021	ECM/-	No
*Helvella lactea*	Helvellaceae	OR142360 (ITS); PQ072264 (*Hsp90*)	IS_241	Forest floor; oak; West Galilee Region, EO and YS, 14 March 2021	ECM/ECM	No
*Helvella lactea*	Helvellaceae	PQ072254 (*Hsp90*)	IS_204	Forest floor; oak; West Galilee Region, EO and YS, 29 January 2021	ECM/-	No
*Helvella lactea*	Helvellaceae	OR142323 (ITS)	IS_18	Forest floor; oak; West Galilee Region, YS, 25 March 2020	ECM/-	No
*Helvella levis*	Helvellaceae	OR141921 (28S); PQ072257 (*Hsp90*)	IS_212	Forest floor; oak, bay; West Galilee Region, EO and YS, 22 February 2021	ECM/ECM	No
*Helvella levis*	Helvellaceae	OR142359 (ITS); OR141929 (28S); PQ072263 (*Hsp90*)	IS_239	Forest floor; bay, oak; West Galilee Region, YS, 14 March 2021	ECM/ECM	No
*Helvella levis*	Helvellaceae	OR141913 (28S); PQ072249 (*Hsp90*)	IS_133	Valley bed; oak; West Galilee Region, EO and YS, 16 March 2021 the closest match is *H. stevensii* which is in the lineage of *H. levis*	ECM/ECM	No
*Helvella levis*	Helvellaceae	PP974222 (ITS)	IS_y-p-19	Forest floor; oak; West Galilee Region, YS, 15 March 2022	ECM/ECM	No
*Helvella levis*	Helvellaceae	OR141896 (28S)	IS_15	Forest floor; oak; West Galilee Region, SM, 22 March 2020	ECM/ECM	No
*Helvella levis*	Helvellaceae	PQ072237 (*Hsp90*)	IS_4	Forest floor; pine, herbs; Carmel Forest, CC, 12 March 2020	ECM/ECM	No
*Helvella levis*	Helvellaceae	OR142321 (ITS); PQ072240 (*Hsp90*)	IS_14	Limestone soil; bryophytes; West Galilee Region, SM, 22 March 2022	ECM/Undetermined	No
*Helvella levis*	Helvellaceae	OR142354 (ITS); PQ072262 (*Hsp90*)	IS_233	Forest floor; *Laurus nobilis*, *Quercus calliprinos*, *Clematis cirrhosa;* West Galilee Region, YS, 07 March 2021	ECM/-	No
*Helvella neopallescens*	Helvellaceae	OR142362 (ITS); OR141930 (28S); PQ072265 (*Hsp90*)	IS_244	Forest floor (pig track); bay, oak; West Galilee Region, YS, 14 March 2021	ECM/ECM	No
*Helvella neopallescens*	Helvellaceae	OR142380 (ITS); PQ072274 (*Hsp90*)	IS_y-p-9	Valley bank; bryophytes; West Galilee Region, YS, 14 March 2022	ECM/ECM	No
*Helvella neopallescens*	Helvellaceae	OR142390 (ITS); PQ072280 (Hsp90)	IS_y-p-24	Forest floor; oak, magnolia; West Galilee Region, YS, 17 March 2022	ECM/ECM	No
*Helvella neopallescens*	Helvellaceae	OR142365 (ITS); OR141931 (28S); PQ072267 (*Hsp90*)	IS_300	Forest floor; oak; Golan Heights, YS, 08 February 2021	ECM/ECM	No
*Helvella neopallescens*	Helvellaceae	PQ072252 (*Hsp90*)	IS_150	Forest floor; bryophytes, oak, ferns; Golan Heights Region, CC, 03 March 2021	ECM/ECM	No
*Helvella poculiformis*	Helvellaceae	OR141922 (28S); PQ072258 (*Hsp90*)	IS_213	Forest floor; oak, bay; West Galilee Region, EO and YS, 22 February 2021	ECM/-	No
*Helvella poculiformis*	Helvellaceae	OR142386 (ITS); PQ072279 (*Hsp90*)	IS_y-p-18	Dry stream bank; bryophyte, oak; West Galilee Region, YS, 15 March 2022	ECM/ECM	No
*Helvella poculiformis*	Helvellaceae	OR142382 (ITS); PQ072278 (*Hsp90*)	IS_y-p-13	Valley bank; bryophytes; West Galilee Region, YS, 14 March 2022	ECM/ECM	No
*Helvella poculiformis*	Helvellaceae	OR141920 (28S); PQ072256 (*Hsp90*)	IS_211	Forest floor; oak; Emek Yizrael Region, YS, 18 February 2021	ECM/-	No
*Helvella retinervis*	Helvellaceae	OR141899 (28S); PQ072241 (*Hsp90*)	IS_26	Forest floor; oak; West Galilee Region, YS, 01 February 2021	ECM/ECM	No
*Helvella retinervis*	Helvellaceae	PQ072276 (*Hsp90*)	IS_y-p-11	Valley bank; bryophytes, oak; West Galilee Region, YS, 14 March 2022	ECM/ECM	No
*Helvella retinervis*	Helvellaceae	OR141894 (28S); PQ072238 (*Hsp90*)	IS_12	Forest floor; oak, pine; West Galilee Region, SM, 22 March 2022	ECM/ECM	No
*Helvella solitaria*	Helvellaceae	OR141926 (28S); PQ072261 (*Hsp90*)	IS_222	Forest floor; bay, oak; West Galilee Region, EO and YS, 23 February 2021	ECM/ECM	No
*Helvella solitaria*	Helvellaceae	PQ072268 (*Hsp90*)	IS_317	Forest floor; Pistacia, oak; West Galilee Region, EO and YS, 13 March 2021	ECM/ECM	No
*Helvella solitaria*	Helvellaceae	OR141915 (28S); PQ072251 (*Hsp90*)	IS_137	Valley bed; bryophyte; West Galilee Region, EO and YS, 16 March 2021	ECM/ECM	No
*Helvella* sp.	Helvellaceae	OR141936 (28S)	IS_y-p-17	Dry stream bank; oak; West Galilee Region, YS, 15 March 2022. Note: Close match to *H. levis* (99%).	ECM/ECM	Maybe
*Helvella* sp. 1	Helvellaceae	PQ072281(*Hsp90*)	IS_503	Forest floor; *Cistus sp*, *Calicotome villosa,* weeds; West Galilee Region, EO and YS, 11 February 2021, Note: close to *H. atra* and *H. hispanica*	ECM/ECM	Maybe
*Helvella* sp.	Helvellaceae	OR141892 (28S)	IS_8	On steep forest wall; bryophytes, oak; West Galilee Region, SM, 22 March 2020	ECM/ECM	Maybe
*Helvella* sp.	Helvellaceae	OR141934 (28S)	IS_y-p-6	Valley floor; bryophytes; West Galilee Region, YS, 14 March 2022, Note: close to *H. pallascens* and *H. vulgate*	ECM/ECM	Maybe
*Humaria* sp. 1	Pyronemataceae	OR142340 (ITS)	IS_124	Forest floor on rich soil; bay; oak; West Galilee Region, EO and YS, 22 March 2021	ECM/ECM	Maybe
*Humaria* sp. 2	Pyronemataceae	OR142325 (ITS); OR141898 (28S)	IS_22	Forest floor; oak, bryophyte; Golan Heights, EO, 22 December 2020, Note: close to *H. hemisphaerica*	ECM/-	Maybe
*Legaliana* sp.1	Pezizaceae	OR142343 (ITS); OR141912 (28S)	IS_132	Valley bed; oak; West Galilee Region, EO&YS, 16 March 2021	ECM/ECM	Maybe
*Legaliana* sp.1	Pezizaceae	OR142377 (ITS)	IS_y-p-2	Valley floor; oak; bryophyte; West Galilee Region, YS, 22 March 2022	ECM/ECM	Maybe
*Legaliana* sp.1	Pezizaceae	PP972214 (28S)	IS_6	Forest floor; oak; West Galilee Region, SM, 14 March 2020, Note: similar to *P. badiofusca*	ECM/Undetermined	Maybe
*Legaliana* sp.2	Pezizaceae	OR142374 (ITS)	IS_1105	Forest floor; oak; West Galilee Region, YS, 31 March 2021	ECM/ECM	Maybe
*Legaliana* sp.2	Pezizaceae	OR142391 (ITS); OR141937 (28S)	IS_y-p-26	Forest floor; oak; West Galilee Region, YS, 20 March 2022	ECM/ECM	Maybe
*Morchella* sp.	Morchellaceae	OR142322 (ITS)	IS_17	Forest floor soil; Lower Galilee Region, LZ and SM, 30 March 2020. Note: ITS sequence is 98.9% similar to *M. vulgaris*; 98.5% similar to *M. elata*;	ECM/-	Maybe
*Otidea adorniae*	Pyronemataceae	OR142341 (ITS)	IS_128	Forest floor; bay, oak; West Galilee Region, EO and YS, 16 March 2021	ECM/ECM	No
*Otidea bufonia*	Pyronemataceae	OR142393 (ITS)	IS_y-p-35	Forest floor; oak; West Galilee Region, YS, 23 November 2023	ECM/-	No
*Paragalactinia* cf. *michelii*	Pezizaceae	OR142357 (ITS)	IS_237	Limestone wall; bryophyte; West Galilee Region, EO and YS, 14 March 2021	ECM/ECM	Yes
*Paragalactinia* cf. *hypoleuca*	Pezizaceae	OR142363 (ITS)	IS_245	Forest floor (pig track); bay, oak; West Galilee Region, EO and YS, 14 March 2021	ECM/-	No
*Paragalactinia* cf. *hypoleuca*	Pezizaceae	OR142364 (ITS)	IS_251	Forest floor; oak, Merom Ha-Galil Region YS, 15 March 2021	ECM/ECM	No
*Paragalactinia* cf. *hypoleuca*	Pezizaceae	OR142349 (ITS); OR141918 (28S)	IS_207	No data	ECM/Undetermined	No
*Paragalactinia* cf. *hypoleuca*	Pezizaceae	OR142389 (ITS)	IS_y-p-23	Dry stream bank; bryophytes; West Galilee Region, YS, 16 March 2022	ECM/ECM	No
*Paragalactinia* cf. *hypoleuca*	Pezizaceae	OR142378 (ITS)	IS_y-p-3	Valley floor; oak, bryophyte; West Galilee Region, YS, 13 March 2022	ECM/ECM	No
*Paragalactinia* cf. *hypoleuca*	Pezizaceae	OR142366 (ITS)	IS_301	Forest floor; oak; Golan Hights Region, EO and SM, 08 February 2021	ECM/ECM	No
*Paragalactinia* cf. *hypoleuca*	Pezizaceae	OR142379 (ITS)	IS_y-p-8	Valley bank; bryophytes, oak; West Galilee Region, YS, 14 March 2022	ECM/ECM	No
*Paragalactinia* cf. *hypoleuca*	Pezizaceae	OR142346 (ITS); OR141916 (28S)	IS_202	Forest floor; oak; West Galilee Region, EO and YS, 21 January 2021	ECM/Undetermined	No
*Paragalactinia succosa*	Pezizaceae	OR141933 (28S)	IS_y-p-5	Valley floor; bryophytes, cyclamen; West Galilee Region, YS, 13 March 20223	ECM/-	Yes
*Paragalactinia* sp.	Pezizaceae	ITS, no GB number	IS-1108	Forest floor; oak, West Galilee Region, YS, 31 March 2021	ECM/-	Maybe
*Paragalactinia* sp. 1	Pezizaceae	OR141928 (28S)	IS_224	Forest floor; bryophytes, West Galilee Region, EO and YS, 23 March 2021	ECM/-	Maybe
*Peziza* cf. *azureoides*	Pezizaceae	OR142318 (ITS)	IS_7	On steep forest wall; bryophytes; West Galilee Region, SM, 22 March 2020	ECM/-	No
*Phaeopezia apiculata*	Pezizaceae	OR142334 (ITS); OR141906 (28S)	IS_101	Trail side; bryophytes, oaks, asparagus, cyclamens, weeds; Naftali Mountains Region, EO, 07 February 2021	ECM/Undetermined	No
*Phylloscypha* *phyllogena*	Pezizaceae	OR142384 (ITS)	IS_y-p-15	Valley slope; bryophytes; West Galilee Region, YS, 15 March 2022	ECM/ECM	No
*Phylloscypha* sp.	Pezizaceae	PP974221 (ITS)	IS_1109	Valley slope; bryophytes; West Galilee Region	ECM/Undetermined	Maybe
*Sarcosphaera* sp. 1	Pezizaceae	OR142319 (ITS)	IS_9	Limestone soil; bryophytes; West Galilee Region, SM, 22 March 2020 Note: close to *S. crassa*	ECM/ECM	Maybe
*Sarcosphaera* sp. 1	Pezizaceae	OR141893 (28S)	IS_10	Forest floor; bryophytes; West Galilee Region, SM, 22 March 2020. Note: close to *S. coronaria*	ECM/-	Maybe
*Sepultariella* *semiimmersa*	Pyronemataceae	OR142388 (ITS)	IS_y-p-22	Dry stream bed; oak, pine; West Galilee Region, YS, 16 March 2022	ECM/ECM	No
*Tarzetta* cf *quercus-ilicis*	Pyronemataceae	OR142352 (ITS); OR141927 (28S)	IS_223	Forest floor; oak; West Galilee Region, EO and YS, 23 February 2021	ECM/ECM	No
*Tarzetta* cf *quercus-ilicis*	Pyronemataceae	OR142348 (ITS); OR141917 (28S)	IS_205	Forest floor; bryophytes; West Galilee Region, YS, 29 January 2021	ECM/Undetermined	No
*Trichophaea* cf. *woolhopeia*	Pyronemataceae	OR142383 (ITS)	IS_y-p-14	Valley bank; bryophytes; West Galilee Region, YS, 14 March 2022, Note: close to *T. woolhopeia* (96.7%)	ECM/Saprobe	Maybe
*Anthracobia* sp.	Pyronemataceae	OR142326 (ITS)	IS_23	Forest floor; burned pine; Carmel Forest Region, SM, 01 January 2021	Saprobe/Saprobe	Maybe
*Daleomyces* sp.	Pezizaceae	OR142342 (ITS)	IS_129	Forest floor; Jerusalem pine, *Pistacia lentiscus,* buckthorn, *Clematis cirrhosa, Smilax aspera;* West Galilee Region, EO and YS, 16 March 2021	Saprobe/ECM	No species
*Daleomyces bicolor*	Pezizaceae	OR142376 (ITS)	IS_y-p-1	Forest floor; oak, pine; West Galilee Region, YS, 02 February 2022	Saprobe/ECM	No
*Elaiopezia* sp.	Pezizaceae	OR142385 (ITS); OR141935 (28S)	IS_y-p-16	Dry stream bank; bryophyte, oak; West Galilee Region, YS, 03 March 2022	Saprobe/ECM	Maybe
*Geopyxis majalis*	Pyronemataceae	OR142339 (ITS)	IS_123	On soil next to path near base of a limestone wall; weeds, bryophytes, cedar; Jerusalem pine; West Galilee Region, EO and YS, 16 March 2021	Saprobe-endophyte/Undetermined	No species
*Geopyxis majalis*	Pyronemataceae	OR142320 (ITS)	IS_11	On forest floor; pine; West Galilee Region, SM, 22 March 2020	Saprobe-endophye/Saprobe	No species
*Melastiza* sp.	Pyronemataceae	OR142329 (ITS); OR141900 (28S)	IS_27	Irrigated hamra soil, compost; West Galilee Region, YS and CC, 01 March 2021	Saprobe/ECM	Maybe
*Peziza varia*	Pezizaceae	OR142338 (ITS)	IS_122	Forest floor, sandy limestone soil, cut branches; fig, cedar; West Galilee Region, EO and YS, 16 March 2021	Saprobe/Saprobe	Yes
*Peziza varia*	Pezizaceae	OR142317 (ITS)	IS_3	Forest floor; Emek Yizrael Region, DL, 07 March 2020	Saprobe/-	Yes
*Peziza* sp. 1 *sensu stricto*	Pezizaceae	OR142324 (ITS); OR141897 (28S)	IS_21	Forest floor, near oak, Golan Heights Region, EO, 24 December 2020, Note: similar to *P. arvernensis*	Saprobe/-	Maybe
*Peziza* sp. 2 *sensu stricto*	Pezizaceae	OR142367 (ITS)	IS_303	Forest floor in clearing, annuals; grass; EO, 08 March 2021, Note: closest to *P. subvesiculosa*	Saprobe/-	Maybe
*Sarcoscypha coccinea*	Sarcoscyphaceae	OR142327 (ITS)	IS_24	Dead oak branch; oak; West Galilee Region, EO and SM & YS, 05 January 2021	Saprobe/Undetermined	Yes
*Sarcoscypha coccinea*	Sarcoscyphaceae	OR142330 (ITS); OR141902 (28S)	IS_30	Dead oak branch; oak; Golan Hights Region, 03 January 2021	Saprobe/-	Yes
*Sarcoscypha coccinea*	Sarcoscyphaceae	OR142345 (ITS)	IS_201	Dead oak branch; oak; West Galilee Region, EO and YS, 21 January 2021	Saprobe/-	Yes
*Sarcoscypha coccinea*	Sarcoscyphaceae	OR142332 (ITS); OR141904 (28S)	IS_32	No data	Saprobe/-	Yes
*Sarcoscypha coccinea*	Sarcoscyphaceae	OR142333 (ITS); OR141905 (28S)	IS_33	No data	Saprobe/-	Yes
*Scutellinia* sp. 1	Pyronemataceae	OR142337 (ITS)	IS_121	Sandy limestone soil in alluvial area next to cut branches; fig, cedar; West Galilee Region, EO and YS, 03 March 2021	Saprobe/-	Maybe
*Scutellinia* sp. 2	Pyronemataceae	OR142316 (ITS)	IS_2	*Scutellinia* sp., OR14 2316 (ITS), dead branch, pine, Gilboa Mountains, DL, 07 March 2020	Saprobe/-	Maybe

Note: a. *Helvella* images were compared with images and descriptions provided in [46,49]. *Sarcosphaera* images were compared with images and descriptions provided in [78].

## References

[1] Hansen K., Pfister D.H. (2006). Systematics of the Pezizomycetes—The operculate discomycetes. Mycologia.

[2] Pfister D.H., Healy R., Zaragoza O., Casadevall A. (2021). Pezizomycetes. Encyclopedia of Mycology.

[3] Hansen K., Sandal S.K., Dissing H. (1998). New and rare species of Pezizales from calcareous woodlands in Denmark. Nord. J. Bot..

[4] Petersen P.M. (1984). The ecology of Danish soil inhabiting Pezizales with emphasis on edaphic conditions. Nord. J. Bot..

[5] Leonardi M., Iotti M., Oddis M., Lalli G., Pacioni G., Leonardi P., Maccherini S., Perini C., Salerni E., Zambonelli A. (2013). Assessment of ectomycorrhizal fungal communities in the natural habitats of *Tuber magnatum* (*Ascomycota*, *Pezizales*). Mycorrhiza.

[6] Ori F., Hall I., Gianchino C., Iotti M., Zambonelli A., Varma A., Tripathi S., Prasad R. (2019). Truffles and morels: Two different evolutionary strategies of fungal-plant interactions in the *Pezizales*. Plant Microbe Interface.

[7] Tedersoo L., May T.W., Smith M.E. (2010). Ectomycorrhizal lifestyle in fungi: Global diversity, distribution, and evolution of phylogenetic lineages. Mycorrhiza.

[8] Lebreton A., Zeng Q., Miyauchi S., Kohler A., Dai Y.C., Martin F.M. (2021). Evolution of the mode of nutrition in symbiotic and saprotrophic fungi in forest ecosystems. Annu. Rev. Ecol. Evol. System..

[9] Nsele N.N., Padayachee T., Nelson D.R., Syed K. (2023). Pezizomycetes genomes reveal diverse P450 complements characteristic of saprotrophic and ectomycorrhizal lifestyles. J. Fungi.

[10] Dirks A.C., Methven A.S., Miller A.N., Orozco-Quime M., Maurice S., Bonito G., Van Wyk J., Ahrendt S., Kuo A., Andreopoulos W. (2025). Phylogenomic insights into the taxonomy, ecology, and mating systems of the lorchel family Discinaceae (Pezizales, Ascomycota). Mol. Phylogenet. Evol..

[11] Selosse M.A., Martos F., Perry B.A., Padamsee M., Roy M., Pailler T. (2010). Saprotrophic fungal mycorrhizal symbionts in achlorophyllous orchid: Finding treasures among the ‘molecular scraps’?. Plant Signal. Behav..

[12] Hibbett D.S., Ohman A., Glotzer D., Nuhn M., Kirk P., Nilsson R.H. (2011). Progress in molecular and morphological taxon discovery in *Fungi* and options for formal classification of environmental sequences. Fungal Biol. Rev..

[13] Truong C., Mujic A.B., Healy R., Kuhar F., Furci G., Torres D., Niskanen T., Sandoval-Leiva P.A., Fernández N., Escobar J.M. (2017). How to know the fungi: Combining field inventories and DNA-barcoding to document fungal diversity. New Phytol..

[14] Bonito G., Smith M.E., Nowak M., Healy R.A., Guevara G., Cazares E., Kinoshita A., Nouhra E., Dominguez L., Tedersoo L. (2013). Historical biogeography and diversification of truffles in the Tuberaceae and their newly identified Southern Hemisphere sister lineage. PLoS ONE.

[15] Kraisitudomsook N., Healy R.A., Pfister D.H., Truong C., Nouhra E., Kuhar F., Mujic A.B., Trappe J.M., Smith M.E. (2019). Resurrecting the genus *Geomorium*: Systematic study of fungi in the genera *Underwoodia* and *Gymnohydnotrya* (*Pezizales*) with the description of three new South American species. Persoonia.

[16] Hobbie E.A., Högberg P. (2012). Nitrogen isotopes link mycorrhizal fungi and plants to nitrogen dynamics. New Phytol..

[17] Hobbie E.A., Sánchez F.S., Rygiewicz P.T. (2004). Carbon use, nitrogen use, and isotopic fractionation of ectomycorrhizal and saprotrophic fungi in natural abundance and ^13^C-labelled cultures. Mycol. Res..

[18] Hobbie E.A., Weber N.S., Trappe J.M. (2001). Mycorrhizal vs saprotrophic status of fungi: The isotopic evidence. New Phytol..

[19] Hobbie E.A., Macko S.A., Shugart H.H. (1999). Insights into nitrogen and carbon dynamics of ectomycorrhizal and saprotrophic fungi from isotopic evidence. Oecologia.

[20] Argiroff W.A., Zak D.R., Pellitier P.T., Upchurch R.A., Belke J.P. (2022). Decay by ectomycorrhizal fungi couples soil organic matter to nitrogen availability. Ecol. Lett..

[21] Talbot J.M., Bruns T.D., Smith D.P., Branco S., Glassman S.I., Erlandson S., Vilgalys R., Peay K.G. (2013). Independent roles of ectomycorrhizal and saprotrophic communities in soil organic matter decomposition. Soil Biol. Biochem..

[22] Henn M.R., Gleixner G., Chapela I.H. (2002). Growth-dependent stable carbon isotope fractionation by basidiomycete fungi: δ13C pattern and physiological process. Appl. Environ. Microbiol..

[23] Hobbie E.A., Rice S.F., Weber N.S., Smith J.E. (2016). Isotopic evidence indicates saprotrophy in post-fire Morchella in Oregon and Alaska. Mycologia.

[24] Healy R.A., Arnold A.E., Bonito G., Huang Y.L., Lemmond B., Pfister D.H., Smith M.E. (2022). Endophytism and endolichenism in Pezizomycetes: The exception or the rule?. New Phytol..

[25] Miyauchi S., Kiss E., Kuo A., Drula E., Kohler A., Sánchez-García M., Morin E., Andreopoulos B., Barry K.W., Bonito G. (2020). Large-scale genome sequencing of mycorrhizal fungi provides insights into the early evolution of symbiotic traits. Nat. Commun..

[26] Selosse M.A., Schneider-Maunoury L., Martos F. (2018). Time to re-think fungal ecology? Fungal ecological niches are often prejudged. New Phytol..

[27] Mayor J.R., Schuur E.A.G., Henkel T.W. (2009). Elucidating the nutritional dynamics of fungi using stable isotopes. Ecol. Lett..

[28] Kadmon R., Danin A. (1997). Floristic variation in Israel: A GIS analysis. Flora.

[29] Levin N., Shmida A. (2007). Determining conservation hotspots across biogeographic regions using rainfall belts: Israel as a case study. Isr. J. Ecol. Evol..

[30] Binyamini N. (2007). Larger Fungi of Israel. Ascomycotina, Basidiomycotina.

[31] Nemlich H., Avizohar-Hershenzon Z. (1972). Pezizales of Israel. I. Morchellaceae and Helvellaceae. Isr. J. Bot..

[32] Barseghyan G.S., Wasser S.P. (2008). Species diversity of operculate discomycetes in Israel. Isr. J. Plant Sci..

[33] Barseghyan G.S., Wasser S.P. (2008). Species diversity of the genera Morchella St. Amans and Helvella L. ex St. Amans (Ascomycota, Pezizales) in Israeli mycobiota. Nova Hedwig..

[34] Barseghyan G.S., Wasser S.P., Volz P.A., Nevo E. (2013). Operculated Discomycetes (Pezizales, Ascomycota) of Israel.

[35] Ferdman Y., Sitrit Y., Li Y.F., Roth-Bejerano N., Kagan-Zur V. (2009). Cryptic species in the *Terfezia boudieri* complex. Antonie Van Leeuwenhoek.

[36] Kagan-Zur V., Roth-Bejerano N. (2008). Studying the brown desert truffles of Israel. Isr. J. Plant Sci..

[37] Morte A., Kagan-Zur V., Navarro-Ródenas A., Sitrit Y. (2021). Cultivation of desert truffles—A crop suitable for arid and semi-arid zones. Agronomy.

[38] Turgeman T., Sitrit Y., Danai O., Luzzati Y., Bustan A., Roth-Bejerano N., Kagan-Zur V., Masaphy S. (2012). Introduced *Tuber aestivum* replacing introduced *Tuber melanosporum*: A case study. Agrofor. Syst..

[39] Masaphy S., Zabari L., Gander G. (2007). Fruiting of morels in Israel. Mushroom Biol. Biotechnol..

[40] Orlofsky E., Zabari L., Bonito G., Masaphy S. (2021). Changes in soil bacteria functional ecology associated with *Morchella rufobrunnea* fruiting in a natural habitat. Environ. Microbiol..

[41] Tietel Z., Masaphy S. (2022). Chemotyping of three *Morchella* species reveals species- and age-related aroma volatile biomarkers. LWT.

[42] Tedersoo L., Smith M.E. (2013). Lineages of ectomycorrhizal fungi revisited: Foraging strategies and novel lineages revealed by sequences from belowground. Fungal Biol. Rev..

[43] Gardes M., Bruns T.D. (1993). ITS primers with enhanced specificity for basidiomycetes—Application to the identification of mycorrhizae and rusts. Mol. Ecol..

[44] White T.J., Bruns T., Lee S., Taylor J., Innis M.A. (1990). Amplification and direct sequencing of fungal ribosomal RNA genes for phylogenetics. PCR Protocols: A Guide to Methods and Applications.

[45] Vilgalys R., Hester M. (1990). Rapid genetic identification and mapping of enzymatically amplified ribosomal DNA from several cryptococcus species. J. Bacteriol..

[46] Skrede I., Carlsen T., Schumacher T. (2017). A synopsis of the saddle fungi (*Helvella*: Ascomycota) in Europe—Species delimitation, taxonomy and typification. Persoonia.

[47] Drummond A.J., Ashton B., Buxton S., Cheung M., Cooper A., Duran C., Field M., Heled J., Kearse M., Markowitz S. Geneious v5.6. http://www.geneious.com.

[48] Schoch C.L., Seifert K.A., Huhndorf S., Robert V., Spouge J.L., Levesque C.A., Chen W., Bolchacova E., Voigt K., Fungal Barcoding Consortium (2012). Nuclear ribosomal internal transcribed spacer (ITS) region as a universal DNA barcode marker for *Fungi*. Proc. Natl. Acad. Sci. USA.

[49] Skrede I., Gonzalvo L.B., Mathiesen C., Schumacher T. (2020). The genera *Helvella* and *Dissingia* (Ascomycota:Pezizomycetes) in Europe—Notes on species from Spain. Fung. Syst. Evol..

[50] Mao N., Xu Y.Y., Zhang Y.X., Huang X.B., Hou C.L., Fan L. (2023). Phylogeny and species diversity of the genus Helvella with emphasis on eighteen new species from China. Fungal Syst. Evol..

[51] Wang X.C., Zhuang W.Y., Zhao R.L. (2023). Species diversity of Helvella lacunosa clade (Pezizales, Ascomycota) in China and description of sixteen new species. J. Fungi.

[52] Yu F.M., Lei L., Luangharn T., Zhao Q., Zhu Y.A. (2023). Four new additions to Helvella (Helvellaceae, Pezizales) from Northern Thailand. Front. Microbiol..

[53] Katoh K., Toh H. (2010). Parallelization of the MAFFT multiple sequence alignment program. Bioinformatics.

[54] Stamatakis A. (2014). RAxML Version 8: A tool for phylogenetic analysis and post-analysis of large phylogenies. Bioinformatics.

[55] Ronquist F., Teslenko M., Van Der Mark P., Ayres D.L., Darling A., Höhna S., Larget B., Liu L., Suchard M.A., Huelsenbeck J.P. (2012). MrBayes 3.2: Efficient Bayesian phylogeneticinference and model choice across a large model space. Syst. Biol..

[56] Darriba D., Taboada G.L., Doallo R., Posada D. (2012). jModelTest 2: More models, new heuristics and parallel computing. Nat. Methods.

[57] Rambaut A., Drummond A.J., Xie D., Baele G., Suchard M.A. (2018). Posterior summarization in Bayesian phylogenetics using Tracer 1.7. Syst. Biol..

[58] Miller M.A., Pfeiffer W., Schwartz T. Creating the CIPRES Science Gateway for inference of large phylogenetic trees. Proceedings of the Gateway Computing Environments Workshop (GCE).

[59] Põlme S., Abarenkov K., Nilsson R.H., Lindahl B.D., Clemmensen K.E., Kauserud H., Nguyen N., Kjøller R., Bates S.T., Baldrian P. (2020). FungalTraits: A user-friendly traits database of fungi and fungus-like stramenopiles. Fungal Divers..

[60] Van Vooren N. (2020). Reinstatement of old taxa and publication of new genera for naming some lineages of the *Pezizaceae* (Ascomycota). Ascomycete.org.

[61] Moreno-Arroyo B., Gómez J., Calonge F.D. (1996). *Pachyphloeus prieguensis*, sp. nov. (Ascomycotina), encontrada en España. Bol. Soc. Micol. Madr..

[62] Healy R.A., Bonito G.M., Trappe J.M. (2009). *Calongea*, a new genus of truffles in the *Pezizaceae* (Pezizales). An. Jard. Bot. Madr..

[63] Sow A., Van Wyk J., Lemmond B., Healy R., Smith M.E., Bonito G. (2024). Effective field collection of Pezizales ascospores for procuring diverse fungal isolates. Diversity.

[64] Dissing H. (1966). The genus Helvella in Europe, with special emphasis on the species found in Norden. Dan. Bot. Ark..

[65] Tedersoo L., Bahram M., Põlme S., Kõljalg U., Yorou N.S., Wijesundera R., Ruiz L.V., Vasco-Palacios A.M., Quang Thu P., Suija A. (2014). Global diversity and geography of soil fungi. Science.

[66] Hansen K., Læssøe T., Pfister D.H. (2002). Phylogenetic diversity in the core group of Peziza inferred from ITS sequences and morphology. Mycol. Res..

[67] Hansen K., LoBuglio K.F., Pfister D.H. (2005). Evolutionary relationships of the cup-fungus genus *Peziza* and *Pezizaceae* inferred from multiple nuclear genes: RPB2, β-tubulin, and LSU rDNA. Mol. Phylogenet. Evol..

[68] Tedersoo L., Suvi T., Larsson E., Kõljalg U. (2006). Diversity and community structure of ectomycorrhizal fungi in a wooded meadow. Mycol. Res..

[69] Hobbie E.A., Agerer R. (2010). Nitrogen isotopes in ectomycorrhizal sporocarps correspond to belowground exploration types. Plant Soil.

[70] Deane-Coe K.K. (2015). Cyanobacteria associations in temperate forest bryophytes revealed by δ^15^N analysis. J. Torrey Bot. Soc..

[71] Korotkin H.B., Swenie R.A., Miettinen O., Budke J.M., Chen K.H., Lutzoni F., Smith M.E., Matheny P.B. (2018). Stable isotope analyses reveal previously unknown trophic mode diversity in the Hymenochaetales. Am. J. Bot..

[72] Ponce Á., Salerni E., D’Aguanno M.N., Perini C. (2023). Wood-decay fungi fructifying in Mediterranean deciduous oak forests: A community composition, richness and productivity study. Forests.

[73] Hansen K., Perry B.A., Dranginis A.W., Pfister D.H. (2013). A phylogeny of the highly diverse cup-fungus family Pyronemataceae (Pezizomycetes, Ascomycota) clarifies relationships and evolution of selected life history traits. Mol. Phylogenet. Evol..

[74] Santiago K.A.A., Ting A.S.Y., Akhtar M.S., Swamy M.K., Sinniah U.R. (2019). Endolichenic fungi from common lichens as new sources for valuable bio-active compounds. Natural Bio-Active Compounds: Volume 1: Production and Applications.

[75] Kohler A., Martin F., Martin F. (2016). The evolution of the mycorrhizal lifestyles—A genomic perspective. Molecular Mycorrhizal Symbiosis.

[76] Khan F.K., Sánchez-García M., Johannesson H., Ryberg M. (2024). High rate of gene family evolution in proximity to the origin of ectomycorrhizal symbiosis in Inocybaceae. New Phytol..

[77] Megersa S., Yohannes Y., Dejene T., Martín-Pinto P. (2024). Natural forests support higher mycological diversity and more edible mushroom species than plantation forests in Ethiopia. For. Int. J. For. Res..

[78] Borovička J., Kolařík M., Halasů V., Perini C., Parker A.D., Gryndler M., Cohen J.D., Hršelová H., Pastorino R., Žigová A. (2024). Taxonomic revision of the genus *Sarcosphaera* (*Ascomycota*: *Pezizales*) in Europe and North America revealed unexpected diversity. Mycol. Prog..

